# Metal Oxide Nano‐Interface Boosting the Deep Ultraviolet Adjustable Noise‐Filtering In‐Sensor Computing

**DOI:** 10.1002/adma.74481

**Published:** 2026-08-06

**Authors:** Zhongshi Ju, Peng Li, Jingsong Yuan, Boyuan Yu, Bin Han, Yusheng Chen, Jiangang Ma, Haiyang Xu, Yichun Liu, Paolo Samorì

**Affiliations:** ^1^ State Key Laboratory of Integrated Optoelectronics Key Laboratory of UV‐Emitting Materials and Technology of Ministry of Education Northeast Normal University Changchun P.R. China; ^2^ University of Strasbourg & CNRS, ISIS & icFRC Strasbourg France; ^3^ School of Chemical Engineering The University of New South Wales Sydney New South Wales Australia; ^4^ College of Materials Xiamen University Xiamen China

**Keywords:** deep ultraviolet, in‐sensor computing, long‐afterglow light‐emitting devices, metal oxide, nano‐interface

## Abstract

Long‐afterglow light‐emitting devices (LALEDs), which combine the capabilities of sensing, memory, processing, and display integration, allow for the simultaneous implementation of optical and electrical in‐sensor computing. However, the inherently low conductivity of conventional deep‐ultraviolet (DUV) responsive materials hinders their use in the DUV‐responsive LALEDs (DUV‐LALEDs). Herein, we demonstrate that sol‐gel‐fractured indium‐magnesium oxide (InMgO) concurrently exhibits ideal nano‐interface for DUV photon absorption and semiconductor crystal for efficient charge transport via hopping, enabling to reach high mobility (0.6 cm^2^ V^−1^ s^−1^), excellent memory dynamic range (70 dB), and responsivity (523.7 A/W). The InMgO‐based DUV‐LALEDs display an electrical and optical post‐synaptic output when irradiated with DUV light. Moreover, hardware‐level noise‐filtering processes to the pre‐synaptic weight are revealed in the DUV‐LALEDs, emerging as the inhibition of light emission due to the insufficient post‐synaptic charge injection from the channel layer. Consequently, an adjustable noise‐filtering in‐sensor computing is successfully achieved by modulating the channel length. By taking advantage of the DUV‐LALED multifunctional nature, fusion‐node reservoir computing networks are employed to accomplish multi‐dimensional recognition tasks, displaying a high recognition accuracy of 99%. These findings demonstrate that the joint materials and devices optimization is a powerful strategy for fabricating cost‐efficient DUV analytical chips.

## Graphical abstract

We show that sol‐gel‐fractured indium–magnesium oxide combines deep‐ultraviolet responsivity, high carrier mobility, and an excellent memory dynamic range. This unique materials platform enables deep‐ultraviolet long‐afterglow light‐emitting devices with multifunctional integration. Furthermore, post‐synaptic charge injection is tuned via the channel distance, providing adjustable hardware‐level noise filtering.

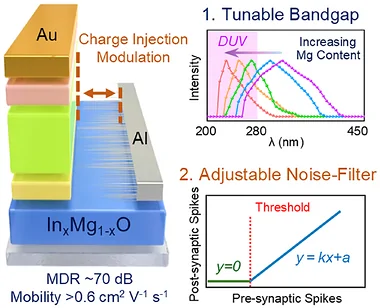

## 1 Introduction

The rapid expansion of artificial intelligence applications has challenged the capabilities of conventional von Neumann computing architectures, in particular due to the intensive data exchange between the separated functional units [1, 2, 3]. Inspired by the human brain, neuromorphic computing architectures proposed an efficient and powerful solution to the processing of big data, which is achieved by incorporating multiple functions into a single device [4, 5, 6]. In this context, artificial retinas have been developed based on active materials exhibiting photo‐induced memory effects, such as the persistent photoconductivity (PPC) effect. Upon light irradiation, these devices retain a photocurrent state even after the stimulus is removed, analogous to the transient memory behavior of retinal neurons. Distinct from conventional photodetectors that lack inherent memory functionality, such unique photo‐memory characteristic facilitates the multifunctional integration toward in‐sensor computing [7, 8, 9]. Recently, long afterglow light‐emitting devices (LALEDs) have been developed, which integrate the sensing, memory, processing and display functions [10, 11]. Pre‐synaptic stimulation of LALEDs powers a post‐synaptic current (PSC) and a post‐synaptic emission (PSE), thereby enabling simultaneous electrical/optical in‐sensor computing, ultimately supporting efficient multitasking processing [12, 13]. Importantly, LALEDs feature a hardware‐level noise‐filtering ability to the pre‐synaptic weight since PSE is observed solely when the PSC surpasses the light‐emission turn‐on current (*I*_On_) [14, 15, 16].

Among optoelectronic artificial synaptic devices with various light wavelength sensory capabilities [17, 18, 19, 20, 21], those involving DUV light leverage the solar‐blind spectral window to achieve exceptionally high signal‐to‐noise ratios. This unique characteristic makes them especially suitable for demanding applications, including flame detection with early warning. The weak DUV emission produced during the initial stages of combustion serves as a distinct signature, which can be reliably identified against a background dominated by visible and infrared radiation [22, 23]. In addition, for secure optical communication, the natural absence of solar DUV at earth's surface creates a low‐noise environment, inherently resistant to interception and ambient interference [24, 25, 26, 27]. However, the incorporation of DUV sensing ability into LALEDs is still challenged by the lack of ultrawide bandgap materials along with superior PPC characteristics. In particular, the conventional ultrawide band gap semiconductors, including the Ga_2_O_3_ and AlGaN, suffer from the trade‐off between a strict manufacturing process and satisfactory conductivity even under DUV irradiation, limiting charge transport which is required to power light‐emitting devices [28, 29, 30]. In contrast, intrinsic n‐type transparent semiconductor metal oxides (In_2_O_3_, ZnO, and SnO_2_) offer outstanding electrical conductivities, but their relatively narrow band gap (< 4 eV) hinders their absorption of DUV light [31, 32, 33]. Therefore, innovative material design strategies that balance wide bandgap characteristics and high electrical conductivities are highly sought after for the development of DUV‐responsive long afterglow light‐emitting devices (DUV‐LALEDs). Moreover, given the demand of diverse noise‐filtering processes, in‐depth investigations of LALEDs with controllable filtering thresholds for the pre‐synaptic weight (*W*_Pre‐Th_) are essential, which can facilitate the adaptive identification of critical signal input.

Herein, we successfully fabricated an unprecedented DUV‐LALED architecture (Figure 1a) integrating sensing‐memory‐computing‐display and possessing adjustable noise‐filtering. The indium‐magnesium oxide (In_x_Mg_1‐x_O) thin film was utilized to emulate the biological retina, wherein DUV‐spiked induced persistent photocarriers were generated at the nano‐interface and underwent hopping to the In_2_O_3_ crystal for efficient charge transport (Figure 1b). Notably, by simply modifying the In/Mg atom ratio, the peak of DUV response wavelength of In_x_Mg_1‐x_O film can be finely tuned, ranging from 320 to 230 nm (Figure 1c). At the same time, the *W*_Pre‐Th_ of DUV‐LALED was adjusted by altering of channel distance between anode and cathode (*D*_AC_), ranging from 60 to 120, 240 and 480 µm (Figure 1d,e). Furthermore, multiphysics coupled simulation revealed that the noise‐filtering process of the LALED is governed by the injection of charges originating from the channel layer into the light‐emitting layer. As a result, we established an equation describing the correlation between *W*_Pre‐Th_ and *D*_AC_, establishing a design guideline for the hardware‐level noise‐filter. The scheme in Figure 1f shows that the neuromorphic architecture based on the multifunctional DUV‐LALED device possesses the advantage of electrical‐optical parallel in‐sensor computing with in situ adjustable noise‐filter, overcoming the limitations of intensive data transmission in conventional von Neumann computing architecture, thus providing a cost‐efficient platform for the multitasking DUV signal analysis.

**FIGURE 1:**
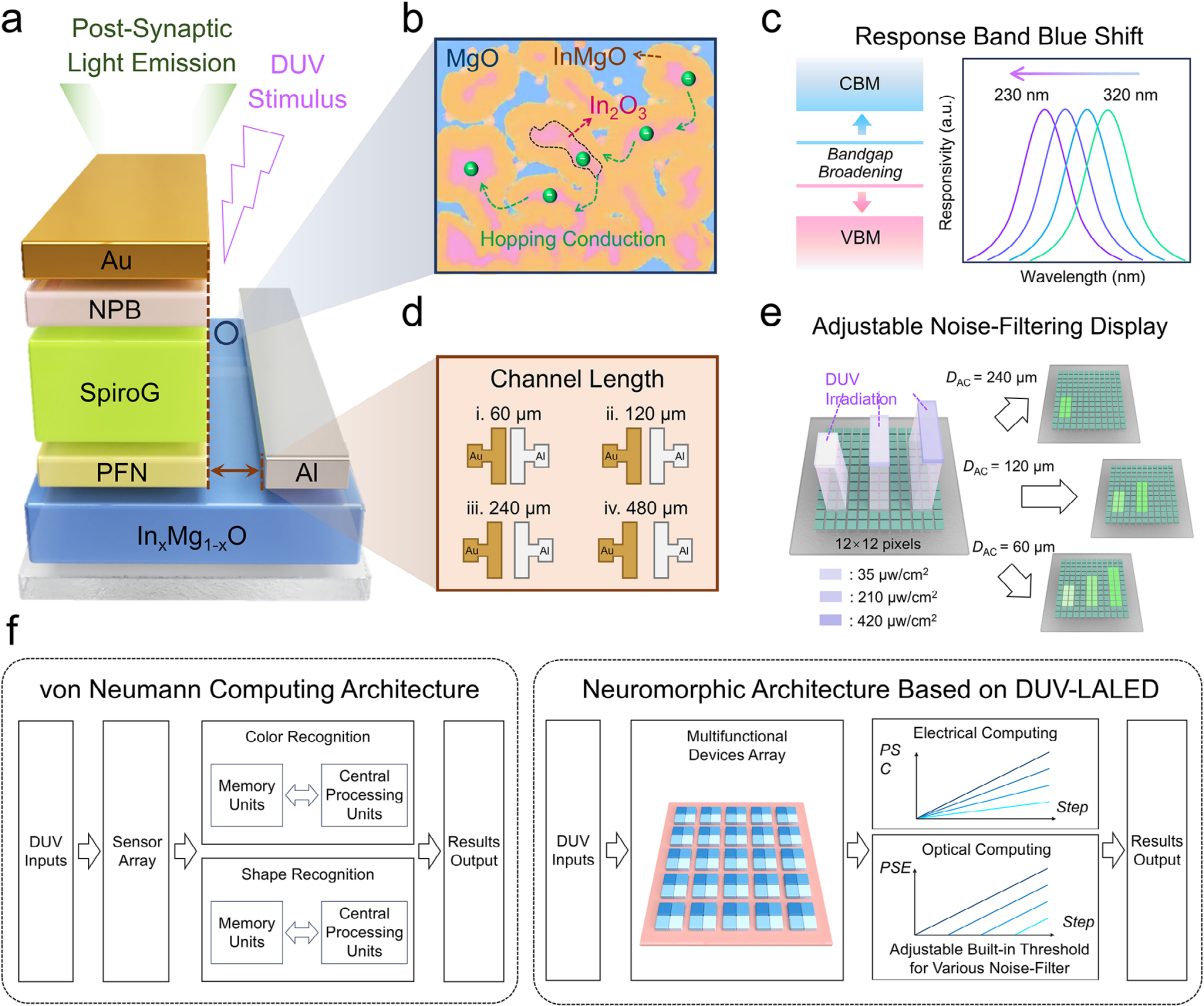
(a) Scheme of DUV‐LALED device architecture. (b) Schematic diagram of In_x_Mg_1‐x_O films with nano‐interface between the semiconductor (In_2_O_3_) and insulator (MgO). (c) Schematic illustration of bandgap broadening and the blue shift of the DUV response band in In_x_Mg_1‐x_O with increasing Mg content. The schematic depiction of (d) *D*_AC_ tuning and (e) adjustable in situ noise‐filter in the devices array with different *D*_AC_, being utilized to classify different DUV strengths. (f) Comparison of von Neumann computing architecture and DUV‐LALED‐based neuromorphic architecture.

## 2 Results and Discussion

### 2.1 Energy Bands and Electronic Structures of In_x_Mg_1‐x_O

The In_x_Mg_1‐x_O thin films were deposited via spin‐coating and subsequently thermally annealed at 450°C in air. Precursors with varying In/Mg atomic ratios, 2:8, 4:6, 5:5, 6:4 and 8:2 were used, yielding films denoted as In_2_Mg_8_O, In_4_Mg_6_O, In_5_Mg_5_O, In_6_Mg_4_O, and In_8_Mg_2_O, respectively. Optical transmission spectra of the films are shown in Figure 2a. With increasing Mg content, the absorption edge blue‐shifts from 320 to 230 nm. The corresponding optical bandgaps of In_2_Mg_8_O, In_4_Mg_6_O, In_5_Mg_5_O, In_6_Mg_4_O, and In_8_Mg_2_O were calculated to be 5.43, 4.98, 4.70, 4.40, and 3.93 eV, respectively. Energy band alignments of the In_x_Mg_1‐x_O films, characterized by x‐ray photoelectron spectroscopy (XPS) and ultraviolet photoelectron spectroscopy (UPS) (Figure S1a,b), are summarized in Figure S1c [34, 35]. After electrode deposition on the In_x_Mg_1‐x_O films, the dark current of devices was measured (Figure S2a). Higher conductivity was observed in the films with greater In content, indicating an increased charge carrier concentration (*n*). The photo‐current of devices under single‐wavelength DUV pulses with different wavelengths were examined (Figure S2b). The normalized responsivity spectra of the In_x_Mg_1‐x_O‐based devices (Figure 2b) revealed a blue shift in the spectral peak from 320 to 230 nm with increasing Mg incorporation, suggesting the potential of DUV in‐sensor computing devices. Furthermore, the field‐effect transistors (FETs) based on In_x_Mg_1‐x_O thin film were fabricated, with their transfer curves being shown in Figure 2c. The mobilities of In_2_Mg_8_O, In_4_Mg_6_O, In_5_Mg_5_O, In_6_Mg_4_O, and In_8_Mg_2_O amounted to 0.2, 0.4, 0.6, 1.1, and 2.0 cm^2^ V^−1^ s^−1^, respectively, significantly exceeding those of conventional DUV absorption materials such as Ga_2_O_3_ and ZnO (Figure S3, Table S1).

**FIGURE 2:**
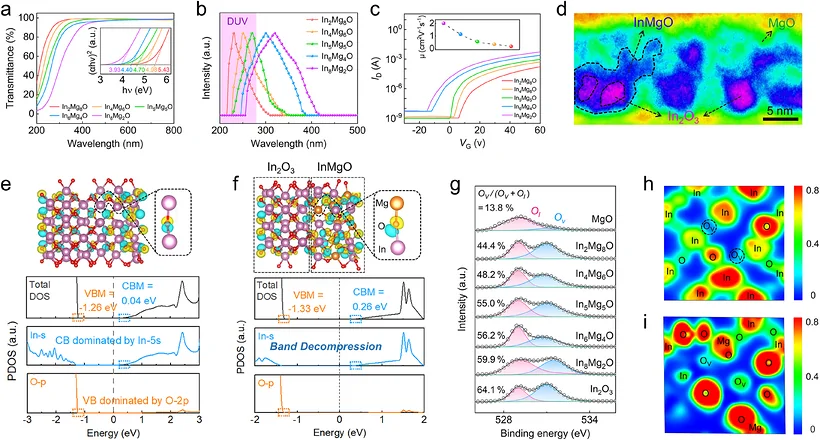
(a) Transmission spectra and corresponding Tauc plot used to extract the optical bandgap (inset) of In_x_Mg_1‐x_O films. (b) Responsivity spectra of In_x_Mg_1‐x_O films‐based two‐terminal devices. (c) Transfer characteristic curves and mobility (shown inset) of In_x_Mg_1‐x_O‐based transistors. (d) HAADF‐STEM image of In_5_Mg_5_O. Calculated atomic structure along with partial charge distribution and the PDOS of oxygen‐deficient (e) In_2_O_3_, (f) In_2_O_3_‐InMgO. The Fermi level was set at 0. (g) O 1s XPS spectra of In_x_Mg_1‐x_O films. ELF contours for (h) In_2_O_3_, (i) In_2_O_3_‐InMgO.

To elucidate the origin of DUV responsivity and superior mobility, the microstructure of In_5_Mg_5_O was investigated using cross‐sectional high‐angle annular dark‐field scanning transmission electron microscopy (HAADF‐STEM). As shown in Figures 2d and S4, three distinct phases were identified: In_2_O_3_ nanocrystals, an MgO matrix, and an InMgO nano‐interface with thickness of ∼10 nm. We propose that the InMgO nano‐interface facilitates DUV photon absorption, while the In_2_O_3_ nanocrystals provide efficient pathways for photo‐triggered electron transport. To validate this hypothesis, density functional theory (DFT) calculations were performed to simulate the electronic structure of In_x_Mg_1‐x_O. Figure 2e,f shows the optimized structures of oxygen‐deficient In_2_O_3_ and its hybrid system with amorphous InMgO. Projected density of states (PDOS) analysis indicates that the valence band is primarily composed of O‐2p orbitals, while the conduction band is dominated by In‐5s orbitals (full PDOS profiles for In, Mg, and O are provided in Figure S5). Compared to pure In_2_O_3_, the conduction band minimum (CBM) of the In_2_O_3_‐InMgO hybrid structure shifts upward by 0.21 eV, and the valence band maximum (VBM) shifts downward by 0.07 eV, resulting in band decompression mainly attributable to In‐s orbital contributions. These computational results are consistent with the experimentally observed optical bandgap widening in Mg‐doped In_2_O_3_ films. The top panel of Figure 2e displays the symmetric differential charge density distribution of In_2_O_3_. In the amorphous InMgO region (Figure 2f), the electron accumulation region (yellow isosurfaces) surrounding each oxygen atom shifts toward adjacent Mg─O bonds, while the electron depletion region (blue isosurfaces) shifts toward In─O bonds. These observations suggest that Mg atoms possess a stronger electron‐withdrawing ability, acting as an acceptor center and thereby reducing the background electron concentration in In_2_O_3_. This finding is consistent with the experimentally observed suppression of oxygen vacancies upon Mg incorporation, as supported by O 1s XPS analysis of In_x_Mg_1‐x_O with varying compositions (Figures 2g and S6) [36]. Thus, the PPC effect of the device is attributed to the oxygen vacancies in the InMgO nano‐interface [37, 38]. Because of this reason, the decayed lifetime of the device decreases with the increasing Mg content (Figure S7). Furthermore, the electron localization function (ELF) profiles reveal that Mg introduction leads to the formation of characteristic localized electron density features (cyan and green regions) at oxygen vacancy sites (indicated by black dashed circles), whereas in pure In_2_O_3_, these regions exhibit a delocalized state (approaching background transparency), as shown in Figure 2h,i. This transition from delocalized to localized electronic states narrows the conduction channels, indicating that Mg incorporation compresses the average carrier hopping transition probability and reduces charge transport capability (Figure S8).

In brief, according to the electronic structure simulations, the formation of InMgO nano‐interface within the thin‐film plays a critical role in modulating photon absorption through band engineering. As the Mg content increases, a trade‐off emerges between bandgap widening and charge‐transport capability. To overcome this limitation, the formation of In_2_O_3_ nanocrystals embedded within the matrix introduces highly conductive domains that serve as efficient hopping pathways for photo‐generated carriers, thereby enabling effective carrier transport across the film. This direct correlation between nanoscale structural features and the resulting electronic behavior underpins the enhanced conductivity relative to conventional homogeneous alloy metal oxides, thereby highlighting the In_x_Mg_1‐x_O thin film as a promising DUV‐sensitive electron channel layer for LALEDs.

### 2.2 DUV Responsive Behavior of In_5_Mg_5_O‐Based Synaptic Device

As illustrated in Figure 3a, the artificial synaptic devices were fabricated by simply depositing aluminum (Al) interdigitated electrodes on the In_x_Mg_1‐x_O thin film. The slow recombination process of DUV‐spiked free electrons was utilized to mimic the Ca^2+^ maintenance process in the biological neuron [39, 40]. The In_5_Mg_5_O composition was selected for the primary device investigations due to its optimal balance between DUV absorption and charge transport mobility. Specifically, several investigated In_x_Mg_1‐x_O thin film compositions, including In_2_Mg_8_O, In_4_Mg_6_O, and In_5_Mg_5_O, exhibit responsivity peaks within the DUV region. Among them, the In_5_Mg_5_O film demonstrates the highest conductivity and largest photocurrent (Figure S2b). This superior charge transport capability is crucial for low‐power in‐sensor computing, resulting in an energy consumption of 2.1 fJ per spike (Figure S9). Consequently, the device performance ranks among the most competitive oxide‐based synaptic systems, as benchmarked in Table S2. Therefore, the In_5_Mg_5_O was ultimately chosen for the main device, meeting the requirements for efficient DUV sensing, charge transport, and photonic memory. And notably, the memory dynamic range (MDR) of the synaptic device can be defined as follows:

(1) $$\mathrm{MDR}=10\times \log\frac{I_{\max}}{I_{\min}}$$

where *I*_max_ and *I*_min_ are extracted from the linear region of PSC curve. As shown in Figures 3b and S10, the *I*_max_/*I*_min_ of In_5_Mg_5_O‐based device amounts to 10^7^ with an excellent MDR of 70 dB, which is beneficial for high operation accuracy in the in‐sensor computing. Meanwhile, the PSC of device increases significantly with prolonged DUV irradiation time, when irradiation time extends from 0.3 to 3 s, leading to the transition from short‐term potentiation (STP) to long‐term potentiation (LTP) (Figure S11). Under constant light intensity and pulse intervals, the photocurrent exhibits cumulative enhancement with the increasing number of pulses (Figure 3c). As plotted in Figure 3d, upon 10 continuous DUV stimulus with stimulus width of 4 s, the PSC of device was triggered to 0.25, 0.42, 0.71, and 1.02 µA, when the DUV power of 8, 16, 24, and 32 µW/cm^2^ was applied, respectively, indicating the spiked‐time‐depend‐plasticity (STDP). These light‐intensity‐dependent, pulse‐accumulative, and relaxation behaviors collectively validate STDP, establishing intrinsic optoelectronic synaptic properties of In_5_Mg_5_O. To gain insight into the mechanism of In_5_Mg_5_O DUV synaptic devices, Kelvin probe force microscopy (KPFM) was measurements were performed for the in situ monitoring of the dynamic surface potential evolution (Figure 3e). Real‐time contact potential difference (CPD) tracking during light switching reveals that CPD stabilized at 10 mV under darkness, then increased to 320 mV upon irradiation, indicating persistent hole accumulation at defect states. After 30 min post‐irradiation, CPD remained at 240 mV. This slow CPD decay kinetics aligns with the device's extended recovery time (> 1 h), confirming hole‐trapping‐induced internal gain as the origin of both high responsivity of 523.7 A/W (under 265 nm light illumination for 5 min) and slow response characteristics. Furthermore, a 4 × 4 vision synaptic array based on In_5_Mg_5_O thin film DUV in an area of 1.5 × 1.5 cm^2^ was fabricated to demonstrate the dynamic imaging behavior (Figures 3f–h and S12). Upon bias voltage of 1 V, the initial device currents of sixteen devices ranged from 30∼80 pA. When subjected to 10 times and 100 times of DUV spikes, the device current increased to 0.9 ∼1.2 and 14 ∼16 µA. The relatively low device‐to‐device variation of ±8% proves the homogeneity of thin‐film, revealing a huge potential for large‐area device fabrication technology.

**FIGURE 3:**
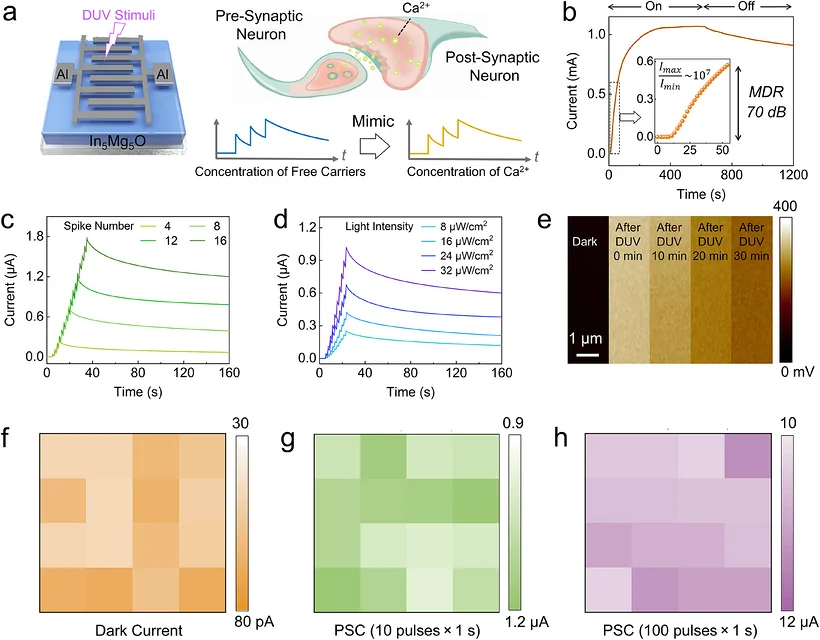
(a) Device architecture of In_5_Mg_5_O‐based DUV synaptic device, which can mimic the Ca^2+^ retention process in the biological neuron by the PPC property. (b) The current variation of the In_5_Mg_5_O‐based DUV synaptic device (with light intensity of 90 µW/cm^2^, under bias voltage of 1 V). (c) The current variation of the In_5_Mg_5_O‐based DUV synaptic device under different spike numbers (with light intensity of 32 µW/cm^2^, under bias voltage of 1 V). (d) The current variation of the In_5_Mg_5_O DUV synaptic device under different DUV light intensity (under bias voltage of 1 V). (e) Surface potential variation of the In_5_Mg_5_O‐based DUV synaptic device before and after 265 nm light illumination. (f) Heat map of dark current for 16 In_5_Mg_5_O‐based DUV synaptic devices. (g) Heat map of PSC for 16 In_5_Mg_5_O‐based DUV synaptic devices after 10 times of DUV stimulations, each with an interval of one second. (h) Heat map of PSC for 16 In_5_Mg_5_O DUV synaptic devices after 100 times of DUV stimulations, each with an interval of 1 s.

### 2.3 Fabrication and Characterization of DUV‐LALEDs

Empowering the in‐sensor computing device with in situ noise‐filtering capability, the light emission function was successfully embedded into a novel DUV‐LALEDs device architecture, with modulable anode‐cathode channel length (Figure 4a). The poly[(9,9‐bis(6′‐(N,N‐diethylamino) hexyl)‐2,7‐fluorene] (PFN), green light‐emitting spiro‐copolymer (SpiroG) and N,N′‐Di(1‐naphthyl)‐N,N′‐diphenyl‐(1,1′‐biphenyl)‐4,4′‐diamine (NPB) were sequentially deposited on the In_5_Mg_5_O, acting as electron transport layer, light emission layer and hole transport layer, respectively, whose chemical structures are portrayed in the Figure 4b. The work mechanism of DUV‐LALED is illustrated in Figure 4c. Upon 265 nm DUV stimuli, electron concentration of In_5_Mg_5_O film increased as a result of a PPC effect, leading the charge horizontally diffused from cathode (aluminum, Al) to the PFN, and subsequently injected into the SpiroG. Hole carriers are injected from the anode (gold, Au) and recombined with electron carrier vertically, resulting in the light emission spread over an area of ∼0.6 mm^2^. In this context, the light emission occurs when sufficient charge transport from Al electrode to Au electrode is present, serving as *I*_On_. The DUV‐LALED with different *D*_AC_ of 60, 120, 240, and 480 µm was fabricated, whose optical microscopy images are shown in Figure 4d. Time‐dependent measurements of these devices are displayed as the PSC and PSE plots reported in Figures 4e,f and S13. If one considers device with *D*_AC_ of 60 µm as an example, upon single DUV stimulus with a strength of 8 µW/cm^2^ and a stimulus width of 4 s, the PSC of device was triggered to 0.4 µA, which fails to fulfill light emission. Prolonging the DUV stimulus to 8, 12, and 16 s, the PSC of devices was widened to 0.9, 1.5, and 1.9 µA, respectively, corresponding with a PSE of 5.6, 7.8, and 11.4 cd/m^2^, respectively. Such results indicate two important issues: (i) An *I*_On_ of 0.5 µA was established as the threshold in the DUV‐LALED, effectively filtering out weak DUV pre‐synaptic weights that failed to active the light‐emission; (ii) both electrical and optical post‐synaptic signals presented STDP. Meanwhile, upon DUV stimulus with various strengths and various number of pulses, the electrical and optical spiked‐strength‐depend‐plasticity (SSDP) and the spiked‐number‐depend‐plasticity (SNDP) were also demonstrated in these devices (Figure S14), confirming their potential for the ANN calculation.

**FIGURE 4:**
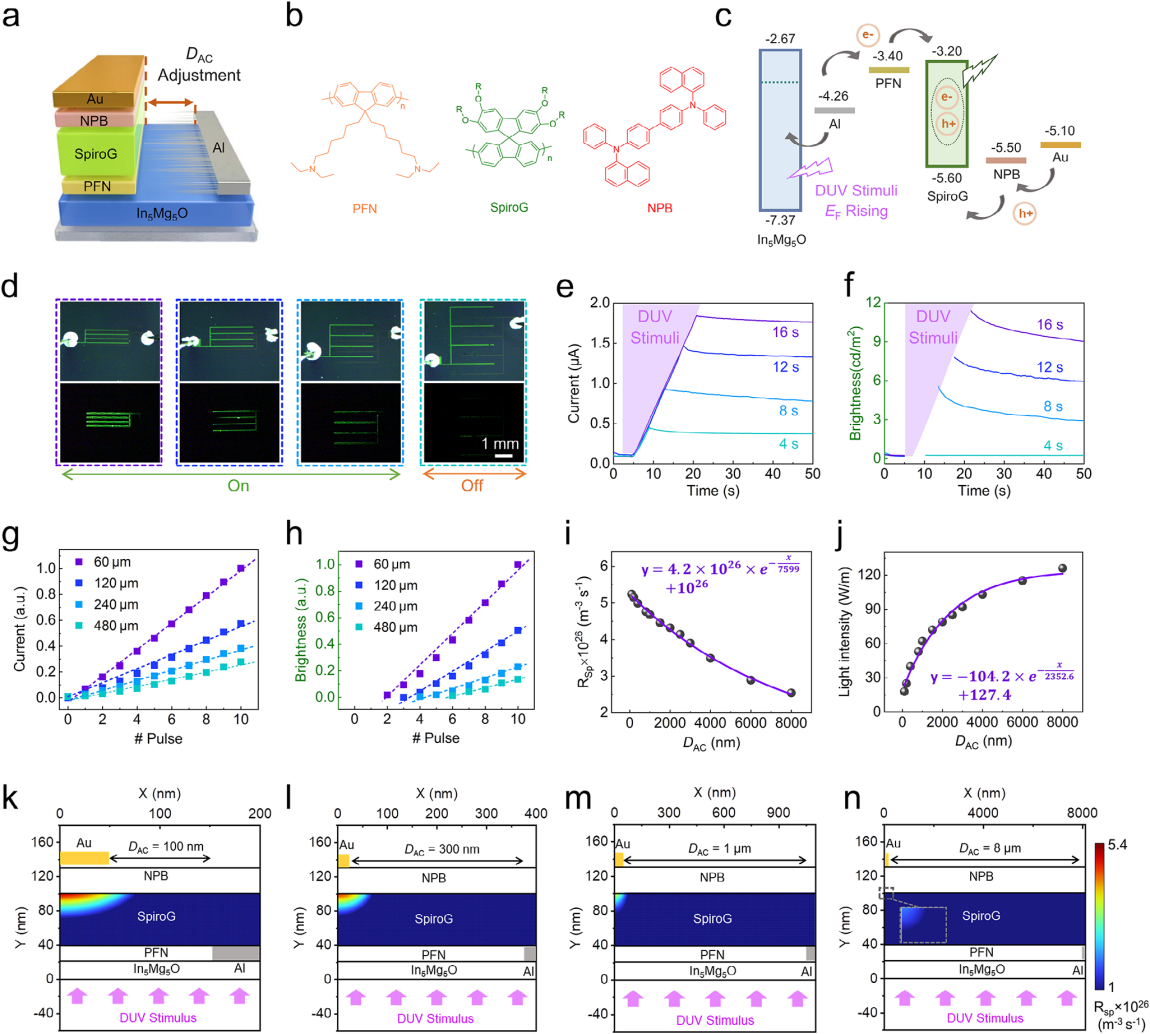
(a) Schematic diagram of DUV‐LALED with adjustable *D*_AC_. (b) Chemical structures of PFN, SpiroG, and NPB. (c) Energy‐level diagram of DUV‐LALEDs representing the hole and electron recombination process. (d) Typical microscopy images of DUV‐LALED with *D*_AC_ of 60, 120, 240, and 80 µm. (e) Current variation of the DUV‐LALED under different DUV stimuli times. (f) Brightness variation of the DUV‐LALED under different DUV stimuli times of 4, 8, 12, and 16 s. (g) Current‐pulse number relationship of DUV‐LALED with *D*_AC_ of 60, 120, 240, and 80 µm. (h) Turn‐on brightness‐pulse number relationship of DUV‐LALED with *D*_AC_ of 60, 120, 240, and 80 µm. COMSOL software simulations of DUV‐LALEDs. (i) *R*_Sp_‐*D*_AC_ relationship (passive *R*_Sp_ decay under constant *n*). (j) Stimulated DUV light intensity‐*D*_AC_ relationship (active DUV light intensity‐tuning for *R*_Sp_ stabilization). *R*_Sp_ spatial distribution for DUV‐LALED with *D*_AC_ of *k*) 100 nm, (l) 300 nm, (m) 1 µm, and (n) 8 µm.

Followingly, PSC and PSE characterization of DUV‐LALED with different *D*_AC_ were conducted when ten continuous DUV stimuli with a 4 s interval were applied (Figure S14). The normalized PSC values and normalized visual brightness values were summarized in Figure 4 g,h, respectively. All DUV‐LALED with diverse *D*_AC_ displayed linear changing memory current states from the first pulse to tenth pulse. Additionally, the *I*_On_ was found to amount to ∼0.5 µA, independent on the *D*_AC_. In contrast, to achieve the *I*_On_, the number of pre‐synaptic spikes (pre‐synaptic weight) is different. Specifically, the turn‐on pulses of PSE were second, third, fourth, and sixth in the DUV‐LALEDs for *D*_AC_ amounting to 60 to 120, 240, and 480 µm, respectively, suggesting the adjustable *W*_Pre‐Th_. Significantly, these results indicate that, in the case of a device with longer channels, stronger pre‐synaptic weight is required to achieve the fixed luminance thresholds. Thus, the noise‐filtering formula of LALED is expressed as the linear rectification function:

(2) $$\begin{array}{c} W_{\mathrm{Post}}=0,\;\mathrm{if}\;W_{\mathrm{Pre}}<W_{\mathrm{Pre}-\mathrm{Th}}\\ W_{\mathrm{Post}}=k\;\left(W_{\mathrm{Pre}}-W_{\mathrm{Pre}-\mathrm{Th}}\right),\;if\;W_{\mathrm{Pre}}\geq W_{\mathrm{Pre}-\mathrm{Th}} \end{array}$$

where the *W*_Pre_ and *W*_Post_ presented as pre‐ and post‐synaptic weight, respectively. In addition, *W*_Pre‐Th_ can be modulated by channel length. To gain insight into the adjustment mechanism of the *W*_Pre‐Th_, optoelectronic field coupling simulations were performed using multiphysics simulations. When using the same functionalized materials and the same device structure, the spontaneous radiative recombination rate (*R*_Sp_) reflects the electroluminescence intensity, whose variation quantitatively scales with brightness output [41, 42, 43]. In these simulations, light emission behavior is intrinsically governed by the recombination efficiency of photogenerated carriers in the DUV‐sensing layer, regulated by *D*_AC_ and the incident DUV light intensity of In_5_Mg_5_O of the DUV‐LALEDs. Therefore, two qualitative simulation issues were carried out, including fixing DUV light intensity and setting *R*_Sp_ (Figure 4i,j). As presented in Figures 4k–n and S15, upon fixing pre‐synaptic DUV light intensity at 1 W/m, various *R*_Sp_ values of devices with different *D*_AC_ were produced. The exponential relationship between *R*_Sp_ and *D*_AC_ was shown in Figure 4i, which can be interpreted as follows. Under constant applied bias, an increase in the *D*_AC_ leads to a significant rise in series resistance. According to Ohm's law, the resulting Ohmic voltage drop across the channel region increases, thereby markedly reducing the effective voltage applied across the light‐emitting layer (SpiroG) that drives carrier injection. This decline in carrier injection efficiency directly causes a reduction in the electron concentration (*n*_0_) and hole concentration (*p*_0_) injected into the SpiroG, as shown in Figures S16 and S17. Since the *R*_Sp_ is proportional to *n*_0_ and *p*_0_ (*R*_Sp_ ∝ *n*_0_·*p*_0_), the decreased carrier concentration ultimately leads to a notable attenuation in the *R*_Sp_. On the other hand, upon fixing *R*_Sp_ of 6 × 10^26^ m^−3^ s^−1^, various light intensities of pre‐synaptic DUV spikes were also observed for the device with different *D*_AC_. As plotted in Figure 4j, the negative correlation between *W*_Pre‐Th_ and *D_AC_* indicates that, to achieve the light emission function in the DUV‐LALEDs, more DUV spikes are required for the device with longer *D*_AC_, owing to need of higher injected carrier concentration (Figures 4j and S18). The fitting curves can be expresses as follows:

(3) $$W_{\mathrm{Pre}-\mathrm{Th}}=a-b\;\times \;e^{-\frac{D_{\mathrm{AC}}}{c}}$$

where the *a, b, c* depends on the employed material and can be extracted from the fitting line. Combined with Equation (2) with (3), the hardware‐level adjustable noise‐filtering process of LALED corresponds to:

(4) $$\begin{array}{ccc} & & \mathrm{if}\;W_{\mathrm{Pre}}<W_{\mathrm{Pre}-\mathrm{Th}},f\left(W_{\mathrm{Pre}}\right)=0\\ & & \mathrm{and}\;\mathrm{if}\;W_{\mathrm{Pre}}\geq W_{\mathrm{Pre}-\mathrm{Th}},f\left(W_{\mathrm{Pre}}\right)=k\left(W_{\mathrm{Pre}}+b\times e^{-\frac{D_{\mathrm{AC}}}{c}}-a\right) \end{array}$$

These results demonstrate that the increase in *D_AC_* is accompanied by an enhancement of the noise‐filtering ability of devices, providing a theoretical guidance for LALED design for in‐sensor computing. Meanwhile, these rationalizations also indicate that when In_x_Mg_1‐x_O with higher In content is used, fewer DUV pre‐synaptic spikes are required for the light emission due to the higher initial *n* value [44, 45, 46].

### 2.4 DUV‐LALED Device Array for Strength Classification

Progressing from image recognition to complex practical applications often requires addressing the challenge of noise interference in different environments. Traditional software denoising methods typically rely on complex multi‐node neural networks for extensive computation. The LALED device in this study achieves for the first time hardware‐level in situ noise filter characteristics realized by *D*_AC_ tuning to directly implement the discriminant function into the light‐emitting layer, ultimately performing noise reduction in stages, as in the proposed architecture reported in Figure 5a,b. As the proof‐of‐concept, the 12 × 12 DUV‐LALED devices array with a *D*_AC_ of 60, 120, and 240 µm were fabricated with a size of 1.28 cm^2^. As recorded in Movies S1–S3, the DUV light with different intensities of 32, 210, and 420 µW/cm^2^ were applied as strong, medium, and weak pre‐synaptic spikes, respectively. As a result, the devices array with a *D*_AC_ of 60, 120, and 240 µm exhibit distinct noise‐filtering processes, which show “all stimulus”, “strong‐medium stimulus” and “strong‐stimulus”, respectively. This hardware‐level integrated processing not only significantly reduces the computational complexity and power consumption of the system but also provides new insights for building more efficient visual perception systems.

**FIGURE 5:**
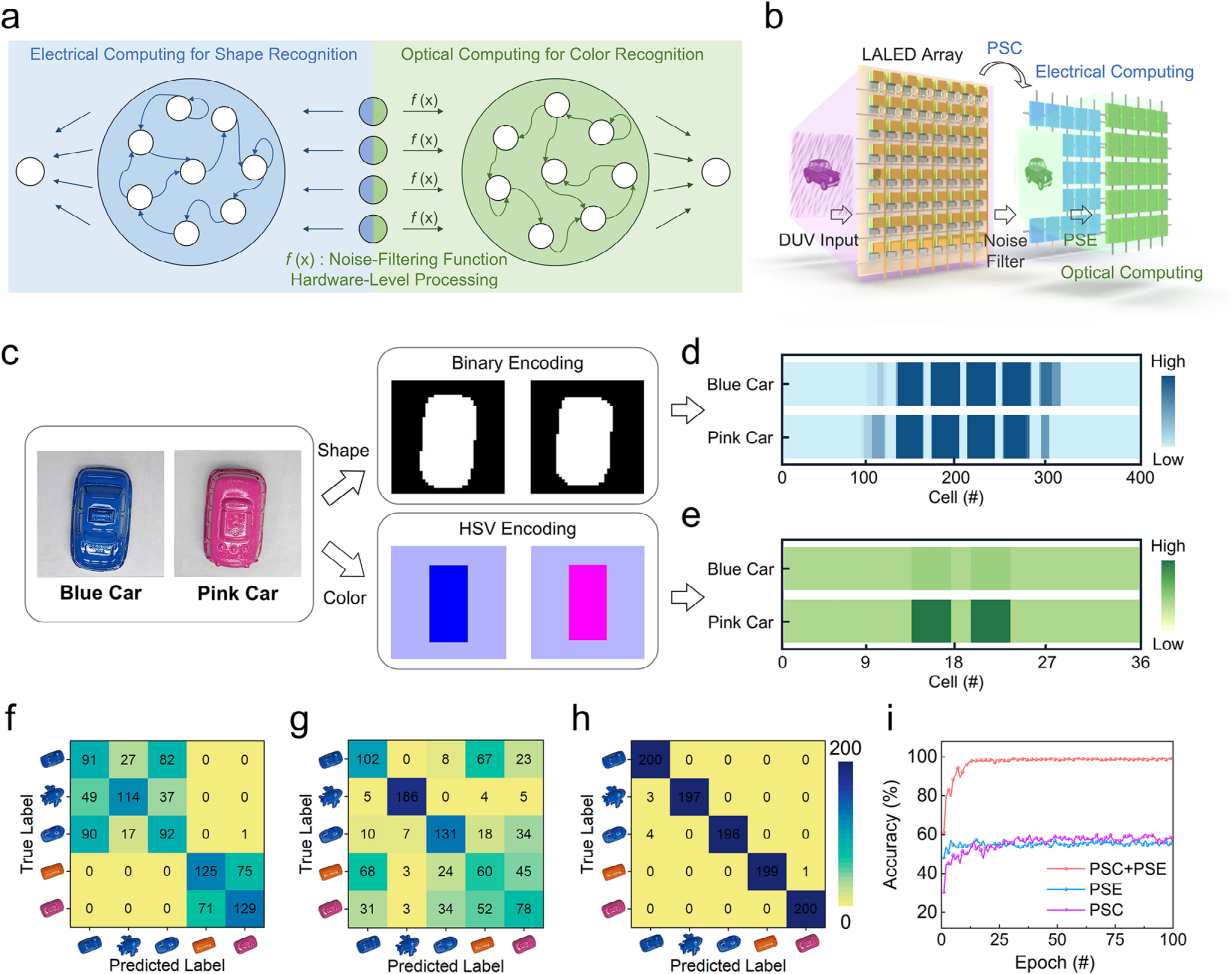
(a) Illustration of a mixed physical node RC system based on LALED, in which the mixed node is constructed from both current and light‐emission post‐synaptic signals. (b) Schematic deception of LALED‐based in‐sensor computing chip architecture. (c) Schematic illustration of the input‐feature encoding process. Shape information is encoded into binary matrices through the PSC‐based reservoir system, whereas color information is encoded into HSV matrices through the PSE‐based reservoir system. (d) Corresponding shape‐feature encoding maps of the blue car and pink car images. (e) Corresponding color‐feature encoding maps of the blue car and pink car images. Confusion matrices for the recognition of blue car, blue plane, blue ship, orange car, and red car images based on (f) PSC‐based RC, (g) PSE‐based RC, and (h) PSC+PSE‐based FNRC. (i) Recognition accuracies of the PSC‐based RC, PSE‐based RC, and PSC+PSE‐based FNRC for the multichannel image dataset.

The reservoir computing (RC) networks have emerged as an efficient framework for image recognition and classification owing to their capability to extract high‐dimensional features from spatiotemporal inputs [47, 48, 49]. Because of its simultaneous integration of DUV sensing and memory capabilities, the as‐prepared DUV‐LALED exhibits strong potential for RC applications. As shown in Figure S19a, 16 distinct PSC and PSE states of the DUV‐LALED were generated by applying four consecutive DUV pre‐synaptic spike inputs ranging from 0000 to 1111, where “1” represents the presence of a DUV spike and “0” denotes the absence of a DUV spike. Based on these 16 reservoir states, object features were subsequently encoded for RC processing. Specifically, shape features were encoded using the 0/1 binary encoding scheme with a 40 × 40 matrix and subsequently were reconstructed into four consecutive pixels across 400 rows. These four‐pixel combinations were further mapped onto 16 reservoir states, thereby yielding a 400‐bit spatiotemporal input sequence. Similarly, color features were encoded through an approximate processing strategy employing a 6 × 6 matrix together with 16 HSV color‐space states, resulting in a 36‐bit spatiotemporal input sequence (Figures 5c and S19b,c). However, conventional RC networks based on a single physical node remain intrinsically limited for object recognition tasks requiring simultaneous discrimination of shape and color features. As illustrated in Figure 5d,e, the encoding maps of the “blue car” and “pink car” exhibit highly similar reservoir encoding maps for shape features but markedly different encoding maps for color features. Conversely, as shown in Figure S19d,e, the “blue car” and “blue plane” possess similar color distributions yet distinct shape‐dependent encoding maps. Consequently, RC systems relying solely on a single‐node architecture (electrical or optical computing, PSC or PSE mode) produced relatively low recognition accuracy. To address these limitations, a fusion‐node RC (FNRC) network based on the DUV‐LALED device was developed. The FNRC simultaneously exploited PSC and PSE computing modes through a parallel‐encoding strategy that synergistically integrates both shape and color information. Benefiting from the complementary feature extraction enabled by the dual‐node architecture, the FNRC achieved highly accurate recognition across all five object categories. After 100 training epochs, the recognition accuracy reached 99% (Figure 5i). The superior performance of the FNRC originates from its ability to process complementary multidimensional information within a single computational pathway, thereby enabling parallel feature extraction with both high accuracy and high operational efficiency. These results clearly demonstrate the considerable potential of the LALED device for multidimensional recognition tasks in in‐sensor computing systems.

## 3 Conclusion

We successfully constructed a DUV responsive sensory‐memory‐processing‐display device, DUV‐LALEDs, which exhibited the adjustable noise‐filtering ability for the in‐sensor computing. Comprehensive studies revealed that the sol‐gel‐derived fractured In_x_Mg_1‐x_O thin film simultaneously exhibits DUV‐induced PPC and high mobility arising from its unique structure composed of InMgO nano‐interfaces and embedded In_2_O_3_ crystalline phase. Specifically, the InMgO nano‐interfaces modulate the band structure while the conductive In_2_O_3_ domains provide efficient hopping pathways for carrier transport. By tuning the In/Mg ratio, it is possible to adjust the DUV light‐responsive peak of In_x_Mg_1‐x_O‐based synaptic device in a range from 320 to 230 nm. Upon incorporating In_5_Mg_5_O into DUV‐LALEDs, both PSC and PSE exhibit STDP when pre‐synaptic spikes of 265 nm DUV light are applied. Light‐emission turn‐on pulses were identified as second, third, fourth, and sixth in the DUV‐LALEDs with 60 to 120, 240, and 480 µm, respectively, proving the adjustable in situ noise‐filter. More importantly, a FNRC networks were successfully constructed by adopting a dual‐encoding strategy that combined optical encoding based on the HSV color space with traditional binary shape encoding. This system can simultaneously extract multi‐dimensional features including color and shape, overcoming the limitations of traditional RC networks that rely on a single physical node. The recognition accuracy of the fusion‐node network for five categories of objects reaches 99% after 100 training sessions, significantly outperforming models relying solely on a single signal. This work highlights the significant application potential of the DUV‐LALED device to accomplish complex tasks through a cost‐efficient approach. Looking ahead, the multifunctional integration of sensing, memory, and display in our DUV‐LALED aligns with the development trend of wearable and implantable platforms, presenting significant potential for next‐generation bio‐integrated optoelectronic systems [50, 51].

## 4 Experimental Section

### 4.1 Materials

Indium nitrate hydrate (In(NO_3_)_3_·*x*H_2_O, 99.9%), magnesium nitrate hydrate (Mg(NO_3_)_2_·*x*H_2_O, 99.9%) and SpiroG were purchased from Sigma–Aldrich. PFN was purchased from Lumtec. NPB and ultra‐flat glass substrates were purchased from Ossila Company. All the materials were used as received without any further purification. Before device fabrication, In(NO_3_)_3_·*x*H_2_O and Mg(NO_3_)_2_·*x*H_2_O were dissolved in 2‐methoxyethanol at a concentration of 0.2 M in the stoichiometric ratio of 2:8, 4:6, 5:5, 6:4, 8:2, and stirred at 50°C overnight; PFN was dissolved in methanol and acetic acid mixing solvent with a concentration of 2 mg/mL; and SpiroG was dissolved in toluene with a concentration of 6 mg/mL.

### 4.2 Device Fabrication

Ultra‐flat glass substrates were cleaned with water, acetone, and alcohol in sequence and dried under N_2_ flow. The substrates were further treated with UV–ozone for 30 min. The In_x_Mg_1‐x_O precursor solution was spin‐coated at 3000 rpm for 60 s on the substrate, followed by drying at 160°C for 5 min, prebaked at 360°C for 30 min and baked at 450°C for 90 min. The Al electrode with a thickness of 40 nm was deposited onto the In_x_Mg_1‐x_O film through shadow masks. An electron transport layer with a thickness of 15 nm was spun from PFN solution, followed by thermal annealing at 150°C for 10 min. The SpiroG layer with a thickness of 50 nm was spun from SpiroG solution, followed by thermal annealing at 150°C for 10 min. The hole transport layer NPB with a thickness of 40 nm was deposited by thermal evaporation. The Au electrode with a thickness of 25 nm was deposited onto the In_x_Mg_1‐x_O film through shadow masks. The width (*W*) and length (*L*) of the channel in LALEDs amounted to 19.2 mm and 60/120/240/480 µm, respectively.

### 4.3 Materials Characterization

Devices were characterized in a dry and nitrogen‐filled glove‐box. The electrical characteristics and basic synaptic functions were measured by a semiconductor parameter analyzer (Keithley 2636). Light emission intensity of the device was measured by a photodiode (HAMAMATSU S3204‐08), which had been previously calibrated by a luminance meter (KONICA MINOLTA, LS‐100). AFM images were recorded with a Bruker Dimension Icon set‐up operating under ambient conditions, in tapping mode. Work functions were probed with the Kelvin probe force microscopy (KPFM, INNOVA INSP‐3). Structural characterizations were accomplished by x‐ray diffraction (XRD) with a Rigaku D/max‐2500 set‐up. XPS investigations and valence band edge spectrum studies were carried out with Thermo Scientific Escalab 250 Xi. The samples for UV/visible absorption spectra measurement were prepared by spin‐coating the solution on clean sapphire, and the spectra were recorded on JASCO V‐500 spectrophotometer. High‐angle annular dark‐field (HAADF) scanning TEM (STEM) investigations were performed by exploiting an electron microscope FEI Talos F200x.

### 4.4 DFT Calculation Methods

All calculations were carried out within the framework of DFT using the Vienna Ab initio Simulation Package (VASP 6.3.2). The generalized gradient approximation (GGA) of the Perdew–Burke–Ernzerhof (PBE) function was used to describe the exchange‐correlation energy. The projected augmented wave (PAW) method and pseudopotentials were used to describe the interactions between valence electrons and ions. To ensure the efficiency of the computational results and parallel computing. A 5 × 5 × 2 k‐point grid under Monkhorst‐Pack is used in the optimization process with the setting truncation energy of 500 V. The lattice parameters and ionic positions of all crystals were fully relaxed, and the convergence criteria for the total energy of all relaxed atoms and the final force were 10^−6^ eV and 0.05 eV/Å, respectively.

### 4.5 Multiphysics Coupled Simulation

Based on COMSOL Multiphysics 6.3, the collaboration of “light field‐to‐electric field simulation” and “electric field‐to‐light field simulation” was achieved by coupling the semiconductor module and the wave optics module. A 2D model of the DUV‐LALED In_5_Mg_5_O (20 nm)/Al/PFN (20 nm)/SpiroG (60 nm)/NPB (30 nm)/Au was built. Ohmic contacts were defined at Al/In_5_Mg_5_O and Au/NPB interfaces, with an applied voltage of 3 V. The wave optics module solved Maxwell's equations under 265 nm DUV illumination (1–128 W m^−1^) and passed the computed initial relative permittivity and electric field amplitude to the semiconductor module, which solved Poisson and drift‑diffusion equations with Fermi–Dirac statistics to obtain carrier transport, recombination, and *R*_Sp_. To investigate the influence of *D*_AC_ on carrier concentration and *R*_Sp_, 12 different *D*_AC_ values ranging from 100 nm to 8 µm were configured. See more details in Supporting Information.

## Conflicts of Interest

The authors declare no conflicts of interest.

## Supporting information




**Supporting file 1**: adma74481‐sup‐0001‐SuppMat.docx.

**Supporting file 2**: adma74481‐sup‐0002‐MovieS1.mp4.

**Supporting file 3**: adma74481‐sup‐0003‐MovieS2.mp4.

**Supporting file 4**: adma74481‐sup‐0004‐MovieS3.mp4.

## Data Availability

The data that support the findings of this study are available from the corresponding author upon reasonable request.

## References

[1] L. Yu , T. Zhang , Z. Wang , et al., “A Fast and Reconfigurable Sort‐in‐Memory System Based on Memristors,” Nature Electronics 8 (2025): 597–609, 10.1038/s41928-025-01405-2.

[2] D. Ielmini and H.‐S. P. Wong , “In‐Memory Computing With Resistive Switching Devices,” Nature Electronics 1 (2018): 333–343, 10.1038/s41928-018-0092-2.

[3] M. Mariantoni , H. Wang , T. Yamamoto , et al., “Implementing the Quantum von Neumann Architecture With Superconducting Circuits,” Science 334 (2011): 61–65, 10.1126/science.1208517. 21885732

[4] D. Ham , H. Park , S. Hwang , and K. Kim , “Neuromorphic Electronics Based on Copying and Pasting the Brain,” Nature Electronics 4 (2021): 635–644, 10.1038/s41928-021-00646-1.

[5] D. Kudithipudi , C. Schuman , C. M. Vineyard , et al., “Neuromorphic Computing at Scale,” Nature 637 (2025): 801–812, 10.1038/s41586-024-08253-8. 39843589

[6] J. W. Lee , J. Han , B. Kang , Y. J. Hong , S. Lee , and I. Jeon , “Strategic Development of Memristors for Neuromorphic Systems: Low‐Power and Reconfigurable Operation,” Advanced Materials 37, no. 19 (2025): 2413916, 10.1002/adma.202413916. 40130789

[7] Z. Zhang , X. Zhao , X. Zhang , et al., “In‐Sensor Reservoir Computing System for Latent Fingerprint Recognition With Deep Ultraviolet Photo‐Synapses and Memristor Array,” Nature Communications 13, no. 1 (2022): 6590, 10.1038/s41467-022-34230-8. PMC9633641 36329017

[8] A. Dodda , D. Jayachandran , A. Pannone , et al., “Active Pixel Sensor Matrix Based on Monolayer MoS_2_ Phototransistor Array,” Nature Materials 21, no. 12 (2022): 1379–1387, 10.1038/s41563-022-01398-9. 36396961

[9] S. Feng , J. Li , L. Feng , et al., “Dual‐Mode Conversion of Photodetector and Neuromorphic Vision Sensor via Bias Voltage Regulation on a Single Device,” Advanced Materials 35, no. 49 (2023): 2308090, 10.1002/adma.202308090. 37813402

[10] Y. Chen , H. Wang , Y. Yao , Y. Wang , C. Ma , and P. Samorì , “Synaptic Plasticity Powering Long‐Afterglow Organic Light‐Emitting Transistors,” Advanced Materials 33, no. 39 (2021): 2103369, 10.1002/adma.202103369. 34369012

[11] W. Kim , K. Lee , S. Choi , et al., “Electrochemiluminescent Tactile Visual Synapse Enabling In Situ Health Monitoring,” Nature Materials 24 (2025): 925–934, 10.1038/s41563-025-02124-x. 39994389

[12] M. Lian , C. Gao , Z. Lin , et al., “Towards Mixed Physical Node Reservoir Computing: Light‐Emitting Synaptic Reservoir System With Dual Photoelectric Output,” Light: Science & Applications 13 (2024): 179, 10.1038/s41377-024-01516-z. PMC11291830 39085198

[13] L. Shan , Q. Chen , R. Yu , et al., “A Sensory Memory Processing System With Multi‐Wavelength Synaptic‐Polychromatic Light Emission for Multi‐Modal Information Recognition,” Nature Communications 14 (2023): 2648, 10.1038/s41467-023-38396-7. PMC10167252 37156788

[14] K.‐T. Chen , P.‐L. Lin , Y.‐C. Huang , S.‐M. Chen , Z.‐S. Liao , and J.‐S. Chen , “Event‐Driven Neuroplasticity and Spiking Modulation in a Photoelectric Neuristor Configured by Threshold Switching Memristor and Optoelectronic Transistor,” Advanced Functional Materials 35, no. 2 (2025): 2412452, 10.1002/adfm.202412452.

[15] Y. Chen , H. Wang , H. Chen , et al., “Li Promoting Long Afterglow Organic Light‐Emitting Transistor for Memory Optocoupler Module,” Advanced Materials 36, no. 27 (2024): 2402515, 10.1002/adma.202402515. 38616719

[16] Y. Chen , H. Wang , F. Luo , V. Montes‐García , Z. Liu , and P. Samorì , “Nanofloating Gate Modulated Synaptic Organic Light‐Emitting Transistors for Reconfigurable Displays,” Science Advances 8, no. 37 (2022): 4824, 10.1126/sciadv.abq4824. PMC9473570 36103533

[17] H. Shao , Y. Li , W. Yang , et al., “A Reconfigurable Optoelectronic Synaptic Transistor With Stable Zr‐CsPbI_3_ Nanocrystals for Visuomorphic Computing,” Advanced Materials 35, no. 12 (2023): 2208497, 10.1002/adma.202208497. 36620940

[18] H. Ahn , Y. Kim , S. Seo , et al., “Artificial Optoelectronic Synapse Featuring Bidirectional Post‐Synaptic Current for Compact and Energy‐Efficient Neural Hardware,” Advanced Materials 37, no. 34 (2025): 70216, 10.1002/adma.70216. 40434208

[19] T. Liu , Z. Yuan , L. Wang , et al., “Chelated Tin Halide Perovskite for Near‐Infrared Neuromorphic Imaging Array Enabling Object Recognition and Motion Perception,” Nature Communications 16 (2025): 4261, 10.1038/s41467-025-59624-2. PMC12059062 40335551

[20] K. Chen , H. Hu , I. Song , et al., “Organic Optoelectronic Synapse Based on Photon‐Modulated Electrochemical Doping,” Nature Photonics 17 (2023): 629–637, 10.1038/s41566-023-01232-x.

[21] Y. Lee , J. Y. Oh , W. Xu , et al., “Stretchable Organic Optoelectronic Sensorimotor Synapse,” Science Advances 4, no. 11 (2018): 7387, 10.1126/sciadv.aat7387. PMC6251720 30480091

[22] S. Kim , F. Nawaz , H. Lee , et al., “Development of Highly Sensitive and Solar Blind Surface Acoustic Wave UV‐C Photodetector Based on the in_2_O_3_/Ta_2_O_5_ Nano‐Heterojunction and Its Interface Electronics,” Sensors and Actuators A: Physical 367, no. 1 (2024): 115027, 10.1016/j.sna.2024.115027.

[23] S. Kim , F. Nawaz , C. Choi , I. Jeon , and K. Lee , “Synthesis and Characterization of High‐Quality Ta_2_O_5_ Nanoparticles for Highly Selective SAW‐Based DUV Sensor and Corona Detection Application,” Advanced Materials Technologies 9, no. 3 (2024): 2301486, 10.1002/admt.202301486.

[24] P. Li , X. Shan , Y. Lin , et al., “Tin Doping Induced High‐Performance Solution‐Processed Ga_2_O_3_ Photosensor Toward Neuromorphic Visual System,” Advanced Functional Materials 33, no. 46 (2023): 2303584, 10.1002/adfm.202303584.

[25] Y. Chen , Y. Li , S. Niu , et al., “High Temperature Resistant Solar‐Blind Ultraviolet Photosensor for Neuromorphic Computing and Cryptography,” Advanced Functional Materials 34, no. 24 (2024): 2315383, 10.1002/adfm.202315383.

[26] Y. Ma , Q. Gao , Y. Li , et al., “Tuning Absorption State and Intermolecular Potential of Organic Semiconductors for Narrowband Ultraviolet Photodetection,” Advanced Materials 37, no. 11 (2025): 2414589, 10.1002/adma.202414589. 39916537

[27] Z. Zhang , X. Zhao , X. Zhang , et al., “In‐Sensor Reservoir Computing System for Latent Fingerprint Recognition With Deep Ultraviolet Photo‐Synapses and Memristor Array,” Nature Communications 13 (2022): 6590, 10.1038/s41467-022-34230-8. PMC9633641 36329017

[28] E. E. Nelson , B. McBennett , T. H. Culman , et al., “Tabletop Deep‐Ultraviolet Transient Grating for Ultrafast Nanoscale Carrier‐Transport Measurements in Ultrawide‐Band‐Gap Materials,” Physical Review Applied 22 (2024): 054007, 10.1103/PhysRevApplied.22.054007.

[29] J. Zhang , J. Willis , Z. Yang , et al., “Direct Determination of Band‐Gap Renormalization in Degenerately Doped Ultrawide Band Gap β‐Ga_2_O_3_ Semiconductor,” Physical Review B 106 (2022): 205305, 10.1103/PhysRevB.106.205305.

[30] Y. Cao , J. W. Pomeroy , M. J. Uren , F. Yang , and M. Kuball , “Electric Field Mapping of Wide‐Bandgap Semiconductor Devices at a Submicrometre Resolution,” Nature Electronics 4 (2021): 478–485, 10.1038/s41928-021-00599-5.

[31] S. Ju , A. Facchetti , Y. Xuan , et al., “Fabrication of Fully Transparent Nanowire Transistors for Transparent and Flexible Electronics,” Nature Nanotechnology 2 (2007): 378–384, 10.1038/nnano.2007.151. 18654311

[32] F. P. Sabino , R. Besse , L. N. Oliveira , S. H. Wei , and J. L. F. Da Silva , “Origin of and Tuning the Optical and Fundamental Band Gaps in Transparent Conducting Oxides: The Case of M_2_O_3_ (M = Al,Ga,In),” Physical Review B 92 (2015): 205308, 10.1103/PhysRevB.92.205308.

[33] M. Han , X. Cai , S. Wei , T. Frauenheim , and P. Deák , “Synergy of Rutile SnO_2_ and TiO_2_ in Optoelectronic Applications: Electronic Structure and Doping Properties of Ti_x_Sn_1‐x_O_2_ Alloys,” Physical Review B 110 (2024): 195203, 10.1103/PhysRevB.110.195203.

[34] E. A. Kraut , R. W. Grant , J. R. Waldrop , and S. P. Kowalczyk , “Precise Determination of the Valence‐Band Edge in X‐Ray Photoemission Spectra: Application to Measurement of Semiconductor Interface Potentials,” Physical Review Letters 44 (1980): 1620, 10.1103/PhysRevLett.44.1620.

[35] X. Hou , Y. Liu , S. Bai , et al., “Pyroelectric Photoconductive Diode for Highly Sensitive and Fast DUV Detection,” Advanced Materials 36, no. 25 (2024): 2314249, 10.1002/adma.202314249. 38564779

[36] X. Hou , X. Zhao , Y. Zhang , et al., “High‐Performance Harsh‐Environment‐Resistant GaO_X_ Solar‐Blind Photodetectors via Defect and Doping Engineering,” Advanced Materials 34, no. 1 (2022): 2106923, 10.1002/adma.202106923. 34626038

[37] M. Lee , W. Lee , S. Choi , et al., “Brain‐Inspired Photonic Neuromorphic Devices Using Photodynamic Amorphous Oxide Semiconductors and Their Persistent Photoconductivity,” Advanced Materials 29, no. 28 (2017): 1700951, 10.1002/adma.201700951. 28514064

[38] S. Jeon , S.‐E. Ahn , I. Song , et al., “Gated Three‐Terminal Device Architecture to Eliminate Persistent Photoconductivity in Oxide Semiconductor Photosensor Arrays,” Nature Materials 11 (2012): 301–305, 10.1038/nmat3256. 22367002

[39] M. A. Sutton and E. M. Schuman , “Dendritic Protein Synthesis, Synaptic Plasticity, and Memory,” Cell 127, no. 1 (2006): 49–58, 10.1016/j.cell.2006.09.014. 17018276

[40] E. Pastalkova , P. Serrano , D. Pinkhasova , et al., “Storage of Spatial Information by the Maintenance Mechanism of LTP,” Science 313 (2006): 1141–1144, 10.1126/science.1128657. 16931766

[41] J. Sobus , F. Bencheikh , M. Mamada , et al., “High Performance P‐and N‐Type Light‐Emitting Field‐Effect Transistors Employing Thermally Activated Delayed Fluorescence,” Advanced Functional Materials 28, no. 28 (2018): 1800340, 10.1002/adfm.201800340.

[42] M. Li , Y. Yang , Z. Kuang , et al., “Acceleration of Radiative Recombination for Efficient Perovskite LEDs,” Nature 630 (2024): 631–635, 10.1038/s41586-024-07460-7. PMC11186751 38811739

[43] X. Tan , D. Dou , L.‐L. Chua , et al., “Inverted Device Architecture for High Efficiency Single‐Layer Organic Light Emitting Diodes With Imbalanced Charge Transport,” Nature Communications 15 (2024): 4107, 10.1038/s41467-024-48553-1. PMC11096390 38750042

[44] J. H. Kang , E. N. Cho , C. E. Kim , et al., “Mobility Enhancement in Amorphous InGaZnO Thin‐Film Transistors by Ar Plasma Treatment,” Applied Physics Letters 102, no. 22 (2013): 222103, 10.1063/1.4809727.

[45] S. H. Kim , M. Kim , J. H. Lee , et al., “Optimizing Length Scalability of InGaZnO Thin‐Film Transistors Through Lateral Carrier Profile Engineering and Negative ΔL Extension Structure,” Advanced Electronic Materials 10, no. 10 (2024): 2400012, 10.1002/aelm.202400012.

[46] J. B. Khurgin , “How to Deal With the Loss in Plasmonics and Metamaterials,” Nature Nanotechnology 10 (2015): 2–6, 10.1038/nnano.2014.310. 25559961

[47] X. Liang , J. Tang , Y. Zhong , B. Gao , H. Qian , and H. Wu , “Physical Reservoir Computing With Emerging Electronics,” Nature Electronics 7 (2024): 193–206, 10.1038/s41928-024-01133-z.

[48] Y. Zhong , J. Tang , X. Li , et al., “A Memristor‐Based Analogue Reservoir Computing System for Real‐Time and Power‐Efficient Signal Processing,” Nature Electronics 5 (2022): 672–681, 10.1038/s41928-022-00838-3.

[49] H. Cai , Z. Ao , C. Tian , et al., “Brain Organoid Reservoir Computing for Artificial Intelligence,” Nature Electronics 6 (2023): 1032–1039, 10.1038/s41928-023-01069-w.

[50] J. Y. Jeon , Q. Chen , S. H. Choi , Y. Chong , I. Jeon , and H. Kim , “Nitride Micro‐LEDs Toward Biointegrated Platforms: Growth, Integration, and Emerging Applications,” ACS Nano 20, no. 16 (2026): 12107–12140, 10.1021/acsnano.5c21282. 41973223

[51] B.‐H. Gong , D. Kim , J. Jeong , et al., “Meniscus Pixel Printing for Contact‐Lens Vision Sensing and Robotic Control,” Advanced Functional Materials 36 (2026): 31981, 10.1002/adfm.202531981.

