# Supplementary material for: Metal Oxide Nano‐Interface Boosting the Deep Ultraviolet Adjustable Noise‐Filtering In‐Sensor Computing
Source: Adv Mater. 2026 Aug 6;38(56):e74481. doi: 10.1002/adma.74481 (PMC13648922; doi:10.1002/adma.74481)
Supplement: Supplementary file 1 [file ADMA-38-e74481-s004.docx]

Supporting Information

Metal oxide nano-interface boosting the deep ultraviolet adjustable noise-filtering in-sensor computing

Zhongshi Ju^⁑^, Peng Li^⁑^, Jingsong Yuan, Boyuan Yu, Bin Han, Yusheng Chen*, Jiangang Ma*, Haiyang Xu*, Yichun Liu, Paolo Samorì*

Z. Ju, P. Li, J. Yuan, J. Ma, H. Xu, Y. Liu

State Key Laboratory of Integrated Optoelectronics, Key Laboratory of UV-Emitting Materials and Technology of Ministry of Education, Northeast Normal University, Changchun, 130024, PR China
E-mail: majg@nenu.edu.cn (J. Ma), hyxu@nenu.edu.cn (H. Xu)

Z. Ju, B. Yu, B. Han, Y. Chen, P. Samorì
University of Strasbourg & CNRS, ISIS & icFRC, 8 allée Gaspard Monge, 67000 Strasbourg, France.

E-mail: samori@unistra.fr (P. Samorì)

B. Yu

School of Chemical Engineering, The University of New South Wales, Sydney, NSW 2052, Australia.

Y. Chen

College of Materials, Xiamen University, Xiamen 361005, P. R. China.

E-mail: yushengchen@xmu.edu.cn (Y. Chen)

**Experimental Section**

*Multiphysics coupled simulation:*

In this study, based on a commercial software COMSOL Multiphysics (version 6.3), the collaboration of “light field-to-electric field simulation” and “electric field-to-light field simulation” was achieved by coupling the semiconductor module and the wave optics module. Two-dimensional models were established to represent the device structures of the DUV-LALEDs, comprising In_5_Mg_5_O/Al/PFN/SpiroG/NPB/Au. The thicknesses of In_5_Mg_5_O, PFN, SpiroG and NPB are 20 nm, 20 nm, 60 nm, and 30 nm, respectively. To simplify the boundary conditions, Ohmic contacts were defined between the Al electrode and both the In_5_Mg_5_O and PFN layers, as well as between the Au electrode and the NPB layer. The applied voltage between Al and Au is set at 3V. Subsequently, the geometric models were discretized to generate quadrilateral-dominant mesh elements, with local refinement introduced in the electrode regions, to enhance the solution accuracy at physical field boundaries and thus to improve overall computational convergence. The wave optics module was used to solve Maxwell’s equations and compute the internal optical field distribution of the device under 265 nm DUV illumination (DUV light intensity: 1-128 W/m). This module transmitted the computed initial relative permittivity and electric field amplitude to the semiconductor Module. Then the semiconductor module coupled and solved the carrier transport and recombination behaviors based on the Poisson and drift-diffusion equations. Specifically, the optical absorption and carrier generation process in the In_5_Mg_5_O layer were configured using the excitation absorption and emission features within the “optical transition” node, the spontaneous emission feature in the same node was utilized to achieve radiative recombination luminescence based on spontaneous emission in the SpiroG layer. Fermi-Dirac statistics were adopted in the semiconductor module to accurately describe carrier distributions under high doping concentrations. To investigate the influence of channel distance between anode and cathode (*D*_AC_) on carrier concentration and the spontaneous radiative recombination rate (*R*_Sp_), twelve different *D*_AC_ values ranging from 100 nm to 8 μm were configured. The computations were ultimately carried out using a frequency-stationary solver, and the output optical intensity was quantitatively evaluated based on *R*_Sp_.

**Figure S1.** a) Valence band edge spectra of In_x_Mg_1-x_O films. b) UPS spectra of In_x_Mg_1-x_O films. c) Energy band arrangement of In_x_Mg_1-x_O films.

The energy difference between the valence band maximum (*E*_v_) and the Fermi level (*E*_F_) of the In_x_Mg_1-x_O films can be determined by fitting the valence band edge spectrum, which are found to be 3.85 eV, 3.57 eV, 3.41 eV, 3.24 eV and 2.99 eV. The absorption spectra of the In_x_Mg_1-x_O films in Figure S1a shows that the optical band gap (*E*_g_) of In_2_Mg_8_O, In_4_Mg_6_O, In_5_Mg_5_O, In_6_Mg_4_O, and In_8_Mg_2_O were determined to be 3.93, 4.40, 4.70, 4.98 and 5.43 eV, respectively. The Fermi level (work function) of In_2_Mg_8_O, In_4_Mg_6_O, In_5_Mg_5_O, In_6_Mg_4_O, and In_8_Mg_2_O were determined to be 3.86, 3.91, 3.96, 4.03, and 4.14 eV, respectively. Thus, the energy band alignments of the In_x_Mg_1-x_O films can be deduced as shown in Figure S1b. The energy band arrangements of In_x_Mg_1-x_O films are summarized in Figure S1c.

**Figure S2.** a) Dark current-bias (*I-V*) characteristic curves of In_2_Mg_8_O, In_4_Mg_6_O, In_5_Mg_5_O, In_6_Mg_4_O, and In_8_Mg_2_O-based synaptic devices. b) Photo current-bias (*I-V*) characteristic curves of In_2_Mg_8_O, In_4_Mg_6_O, In_5_Mg_5_O, In_6_Mg_4_O, and In_8_Mg_2_O-based synaptic devices under 230 nm, 250 nm, 265 nm, 290 nm and 320 nm light irradiation with light intensity of 8 μW/cm^2^ for 60 s, respectively. Responsivity of c) In_2_Mg_8_O-based synaptic device, d) In_4_Mg_6_O-based synaptic device, e) In_5_Mg_5_O-based synaptic device, f) In_6_Mg_4_O-based synaptic device and g) In_8_Mg_2_O-based synaptic device, respectively.

The In_5_Mg_5_O composition was selected for primary device investigations owing to its optimal balance between DUV absorption and charge transport mobility. Moreover, to quantify the spectral selectivity, we calculated the rejection ratios *R* (λ_peak_)/*R* (λ) from the responsivity curve (Figure S2e). The device exhibits a peak responsivity at 265 nm (UVC). The measured rejection ratios are: *R*_peak_/*R*_320 nm_ (UVC/UVB) = 9.4, *R*_peak_/*R*_360 nm_ (UVC/UVA) = 9.5×10^4^, and *R*_peak_/*R*_400 nm_ (UVC/visible) = 9.5×10^4^. The high rejection ratio against longer wavelengths (UVA and visible), which is competitive with reported photodetectors, demonstrated the device’s outstanding ability to suppress interference from the solar-rich UVA and visible background.^[S1-S6]^

**Figure S3.** Comparison of mobilities of wide bandgap semiconductor-based synaptic devices.^[S7-S20]^

The achieved field-effect mobility (0.17-2.02 cm^2^ V^-1^ s^-1^) for our solution-processed In_x_Mg_1-x_O film is meaningful within the landscape of wide-bandgap semiconductors. A clear trade-off between bandgap and mobility is observed across the metal oxide materials (fitting line). This phenomenon can be explained as follows: an increased *E*_g_ commonly leads to a larger carrier effective mass (*m*^*^) according to the equation S1.^[S21,S22]^

$\frac{m_{e}}{m^{*}}=1+\frac{2}{m_{e}+E_{g}}\left| \left\langle\nu| p_{x} | c \right\rangle\right|^{2}$	(S1)

where *m*_e_ is the free electron mass, *p*_x_ is the momentum operator in the x direction, $\left. \left| c \right. \right\rangle$is the state at the conduction band minimum, and $\left. v \right\rangle$ is the light-hole state at the valence band maximum. Furthermore, according to the Drude equation

$\mu=\frac{e\tau}{m^{*}}$	(S2)

where *μ* is mobility, *e* is charge, and *τ* is scattering time, a larger *m*^*^, indicates that an increased effective mass directly reduces mobility, thereby explaining the observed trend. Notably, within the UVC spectral range, our In_x_Mg_1-x_O film (red star) stands out as a competitive material, exhibiting higher mobility than many Ga_2_O_3_-based counterparts, successfully balancing a wide bandgap for UVC selectivity and sufficient conductivity for device operation.

**Figure S4.** a) STEM image of In_5_Mg_5_O film fabricated on the sapphire substrate. b) Selected area electron diffraction pattern of amorphous MgO. c) Selected area electron diffraction pattern of polycrystal In_2_O_3_. d) XRD patterns of In_2_Mg_8_O, In_4_Mg_6_O, In_5_Mg_5_O, In_6_Mg_4_O, and In_8_Mg_2_O films.

The bright-field scanning transmission electron microscopy (HAADF-STEM) image exhibits distinct contrast variations, where the dark regions correspond to In_2_O_3_ with a higher atomic mass, while the light regions correspond MgO with a lower atomic mass. This contrast clearly reveals a structure where In_2_O_3_ is embedded within the MgO matrix. Selected area electron diffraction (SAED) analysis of the MgO region confirms it is amorphous. In contrast, the SAED pattern from the In_2_O_3_ region shows concentric diffraction rings that are well-indexed to the (104) and (012) planes of crystalline In_2_O_3_. Furthermore, X-ray diffraction (XRD) results indicate the presence of only the crystalline In_2_O_3_ phase, providing additional evidence that the MgO matrix is amorphous.

**Figure S5.** PDOS of oxygen-deﬁcient a) In_2_O_3_ and b) In_2_O_3_-InMgO.

To provide in-depth insights into electrical properties of the oxygen-deﬁcient In_2_O_3_-InMgO, we performed DFT calculations. The conduction band minimum (CBM) and valence band maximum (VBM) can be deduced from the DOS.^[S23-S26]^

**Figure S6.** a) Mg 2p XPS spectra of MgO, In_2_Mg_8_O, In_4_Mg_6_O, In_5_Mg_5_O, In_6_Mg_4_O, and In_8_Mg_2_O films. b) In 3d XPS spectra of In_2_Mg_8_O, In_4_Mg_6_O, In_5_Mg_5_O, In_6_Mg_4_O, In_8_Mg_2_O and In_2_O_3_ films.

The surface chemical states and elemental composition of the In_x_Mg_1-x_O films were recorded by using XPS. For the Mg 2p XPS, the peak located at about 47 eV is assigned to the Mg-O.^[S27-S29]^ For the In 3d XPS, two peaks located at 441 eV and 449 eV are assigned to the In-O.^[S30-S32]^ As the In/Mg ratio varied, the XPS peak positions of In-O and Mg-O exhibited directional shifts, the binding energy of the In-O bond shifted by 0.75 eV towards higher energy, whereas the binding energy of the Mg-O bond shifted by 0.68 eV towards lower energy. This opposite-direction shift in binding energies indicates diffusive bonding among In, Mg, and O atoms, forming the InMgO alloy interface and driving directional electron flow from the In-O bond region to the Mg-O bond region, generating a dipole moment pointing from In-O to Mg-O.

**Figure S7.** The Corresponding decay curve (hollow circles) and fitting line of post-synaptic current of In_2_Mg_8_O, In_4_Mg_6_O, In_5_Mg_5_O, In_6_Mg_4_O, and In_8_Mg_2_O-based synaptic devices.

The lifetime (*τ*) of the devices can be also determined from the time-resolved decay of post-synaptic current in the exponential decay equation: *L*(t) = *L*_0_e^-^*^t^*^/τ^ + *L*_∞_, where *L*_0_ is the initial current at starting time (*t* = 0).^[S33,S34]^ The τ of In_2_Mg_8_O, In_4_Mg_6_O, In_5_Mg_5_O, In_6_Mg_4_O, and In_8_Mg_2_O-based synaptic devices can be deduced to be 520 s, 384 s, 380 s, 274 s and 193 s, respectively.

**Figure S8.** a) Total resistivity (*R*_T_) of Al/In_x_Mg_1-x_O/Al devices. b) Contact resistivity (*R*_C_) of Al/In_x_Mg_1-x_O interfaces.

The contact resistivity between metal and semiconductor was measured using the Transmission Line Model.^[S35-S37]^ As the In content increases, the overall resistance of the In_x_Mg_1-x_O films decreases significantly. Although the initial dark conductivity of these films is relatively low, the presence of highly conductive In_2_O_3_ nanocrystals creates efficient hopping pathways for photo-generated carriers, enabling enhanced charge transport despite the matrix's limited conductivity.

**Figure S9.** The current variation of the In_5_Mg_5_O DUV synaptic device (with light intensity of 1.5 mW/cm^2^, under bias voltage of 10 μV).

Energy consumption per spike is a key metric for in-sensor computing applications. The energy per spike for our In_5_Mg_5_O synaptic device was calculated from the photocurrent under pulsed DUV illumination at a 10 μV bias, using the formula *E = V × I × t* (where *V* is the applied voltage, *I* is the photocurrent, and *t* is the spike time). Our device consumes 2.1 fJ per spike, significantly outperforming many reported works based on Ga_2_O_3_, IGZO and other devices operating at pJ or nJ levels (Table S2). This low energy consumption originates from the low operating voltage and the high photoresponsivity of In_5_Mg_5_O. Such an ultralow energy proves the great potential of our devices for building energy-efficient in-sensor computing and large-scale neuromorphic systems.

**Figure S10.** The logarithmic current variation of the In_5_Mg_5_O DUV synaptic device (with light intensity of 90 μW/cm^2^, under bias voltage of 1 V).

**Figure S11.** The current variation of the In_5_Mg_5_O DUV synaptic device under diﬀerent interval time (with light intensity of 32 μW/cm^2^, under bias voltage of 1 V).

**Figure S12.** a) The current variation of the In_5_Mg_5_O DUV synaptic device under 10 times of 265 nm DUV stimulations, each with an interval of one second. b) The current variation of the In_5_Mg_5_O DUV synaptic device under 100 times of 265 nm DUV stimulations, each with an interval of one second.

The variation in the pre-stimulation dark current (30 - 80 pA) arises from the inherent sensitivity of pA-level measurements to environmental noise. Crucially, the DUV-induced photocurrent exceeds 1 μA, which is several orders of magnitude higher and therefore dominates the overall response, rendering it largely insensitive to the same noise sources. The reported ±8% variation in the saturated PSC thus accurately reflects the intrinsic device-to-device uniformity of the optoelectronic response.

**Figure S13.** The current variation a) and the brightness variation b) of the DUV-LALED with *D*_AC_ of 120 μm under diﬀerent DUV stimuli times of 4 s, 8 s, 12 s and 16 s. The current variation c) and the brightness variation d) of the DUV-LALED with *D*_AC_ of 240 μm under diﬀerent DUV stimuli times of 4 s, 8 s, 12 s, and 16 s. The current variation e) and the brightness variation f) of the DUV-LALED with *D*_AC_ of 480 μm under diﬀerent DUV stimuli times of 4 s, 8 s, 12 s, and 16 s.

**Figure S14.** The current variation a) and the brightness variation b) of the DUV-LALED with *D*_AC_ of 60 μm under diﬀerent DUV intensities of 8, 12, 18 and 30 μW/cm^2^. The current variation c) and the brightness variation d) of the DUV-LALED with *D*_AC_ of 120 μm under diﬀerent DUV intensity of 8, 12, 18 and 30 μW/cm^2^. The current variation e) and the brightness variation f) of the DUV-LALED with *D*_AC_ of 240 μm under diﬀerent DUV intensities of 8, 12, 18 and 30 μW/cm^2^. The current variation g) and the brightness variation h) of the DUV-LALED with *D*_AC_ of 480 μm under diﬀerent DUV intensities of 8, 12, 18 and 30 μW/cm^2^.

**Figure S15.** *R*_Sp_ spatial distribution for DUV-LALEDs.

**Figure S16.** Electron concentration (*n*_0_) spatial distribution for DUV-LALEDs.

The simulation results indicate that electrons primarily accumulate on the In_5_Mg_5_O and PFN layers. The high electron concentration in the In_5_Mg_5_O layer stems from photogenerated electrons produced by 265 nm light excitation, while the PFN layer efficiently transports electrons to the upper materials due to its band alignment with In_5_Mg_5_O. As SpiroG layer is designated as the electron-hole recombination region, the electron concentration is low because it specifically refers to the steady-state concentration of non-equilibrium carriers that have not undergone recombination (including luminescent recombination).

**Figure S17.** Hole concentration (*p*_0_) spatial distribution for DUV-LALEDs.

The simulation results indicate that holes primarily accumulate in the In₅Mg₅O and NPB layers. The high hole concentration in the In₅Mg₅O layer originates from photogenerated holes produced by 265 nm light excitation, while the NPB layer, functioning as a hole transport layer, efficiently delivers holes to the underlying materials. It is noted that the extremely low hole concentration in the PFN layer confirms its role not only as an electron transport layer but also as a hole blocking layer.

**Figure S18.** *R*_Sp_ spatial distribution for DUV-LALEDs with fixing *R*_Sp_.

**Figure S19.** a) PSC and PSE reservoir states of the LALEDs under pre-synaptic spike inputs ranging from 0000 to 1111. b) Color encoding based on 16 states derived from HSV color-space partitioning. c) Schematic illustration of the input-feature encoding process. Shape information is encoded into binary matrices through the PSC-based reservoir system, whereas color information is encoded into HSV matrices through the PSE-based reservoir system. d) Corresponding shape-feature encoding maps of the blue car and blue plane images. e) Corresponding color-feature encoding maps of the blue car and blue plane images.

**Figure S20.** Electroluminescence (EL) spectra of SpiroG of DUV-LALEDs with *D*_AC_ of 60 μm, 120 μm, 240 μm and 480 μm, respectively.

The EL spectra confirm spectral uniformity across all *D*_AC_ with identical 510 nm emission peaks, eliminating spectral distortion concerns.

**Figure S21.** a) XRD patterns of In_5_Mg_5_O films under annealing temperatures of 350 ℃, 450 ℃, 550 ℃ and 650 ℃, respectively. b) The current variation of the In_5_Mg_5_O DUV synaptic devices (with light intensity of 32 μW/cm^2^, under bias voltage of 1 V). c) Current-bias (*I-V*) characteristic curves of In_5_Mg_5_O DUV synaptic devices.

To elucidate the crystallization process and nano-heterostructure evolution, In_5_Mg_5_O films were annealed at different temperatures (350, 450, 550, and 650 °C). As shown in Figure S21a, the film annealed at 350 °C remains largely amorphous, whereas films annealed at 450-650 °C exhibit distinct diffraction peaks corresponding to crystalline In_2_O_3_. Notably, the peak positions and widths show negligible variation with further increasing annealing temperature, suggesting that the crystallinity of In_2_O_3_ does not improve significantly beyond 450 °C. In contrast, no characteristic MgO diffraction peak is observed even at 650 °C, indicating that the MgO phase remains amorphous throughout the investigated temperature range. Combined with the electrical characterization in Figure S21b,c, devices annealed at 350 °C exhibit extremely low dark current and weak PPC behavior, which can be attributed to incomplete removal of residual solvents and insufficient formation of conductive In_2_O_3_ pathway. Although slightly enhanced conductivity and photocurrent are obtained at higher annealing temperatures (550 and 650 °C), excessively high processing temperatures may limit practical device integration and large-scale fabrication. Therefore, an annealing temperature of 450 °C is selected as the optimal condition for realizing the In_x_Mg_1-x_O thin films in this work.

**Figure S22.** a) The current variation of the In_5_Mg_5_O DUV synaptic devices under test temperatures of 298 K, 313 K, 333 K and 353 K, respectively (with light intensity of 32 μW/cm^2^, under bias voltage of 1 V). b) Dark current-bias (*I-V*) characteristic curves of In_5_Mg_5_O DUV synaptic devices under test temperatures of 298 K, 313 K, 333 K and 353 K, respectively. c) The current variation of the In_5_Mg_5_O DUV synaptic devices under test humidities of 20% RH, 40% RH, 60% RH and 80% RH, respectively (with light intensity of 32 μW/cm^2^, under bias voltage of 1 V). d) Dark current-bias (*I-V*) characteristic curves of In_5_Mg_5_O DUV synaptic devices under test humidities of 20% RH, 40% RH, 60% RH and 80% RH, respectively.

To evaluate environmental stability, devices were tested under controlled temperature (298, 313, 333, and 353 K) and relative humidity (20%, 40%, 60%, and 80% RH). As shown in Figure S22a,b, the device current increases with temperature, indicating the enhanced thermally activated charge hopping. In Figure S22c,d, the PPC behavior is influenced significantly by the humidity. The decay rate of PSC enhances with rising humidity, which can be attributed to accelerated recombination of trapped charges facilitated by interactions with adsorbed water molecules.^[S38–S41]^ Therefore, effective encapsulation is essential for reliable device operation in practical applications.

**Figure S23.** The current variation of the In_5_Mg_5_O DUV synaptic devices (with light intensity of 90 μW/cm^2^, under bias voltage of 1 V).

The photocurrent of the device was monitored under constant 265 nm DUV irradiation for 24 h. After reaching saturation, the photocurrent remained stable within a narrow range of 0.98-1.10 mA, demonstrating excellent long-term operational stability of In_5_Mg_5_O DUV synaptic devices under continuous illumination.

**Figure S24.** EDS mapping images of the In_5_Mg_5_O film a) before and c) after 265 nm DUV irradiation for 24 h. Corresponding integrated spectrum of the In_5_Mg_5_O film b) before and d) after 265 nm DUV irradiation for 24 h.

Energy dispersive spectrometer (EDS) elemental mapping and integrated spectra were obtained on the same In_5_Mg_5_O film before and after the 24 h DUV irradiation. The In:Mg atomic ratio remained nearly unchanged (1:0.96 and 1:0.98), indicating that the elemental composition is well preserved. These results confirm that only negligible compositional variation occurs under prolonged DUV exposure, suggesting high material stability under continuous irradiation.

**Figure S25.** The current variation of the In_5_Mg_5_O DUV synaptic devices that measured daily over 14 days (spiked by the single DUV pulse with light intensity of 32 μW/cm^2^, under bias voltage of 1 V).

The current-time (*I*-*t*) curves recorded on different days are nearly identical, indicating excellent device repeatability under DUV irradiation.

**Table S1. Comparison of mobilities of wide bandgap semiconductor-based synaptic devices.**

| Material | Bandgap  (eV) | Mobility  (cm^2^ V^-1^ s^-1^) | Reference |
| --- | --- | --- | --- |
| ZnO | 3.60 | 0.15 | S7 |
| ZnO | 3.60 | 1.60 | S8 |
| ZnO | 3.60 | 3×10^3^ | S9 |
| ZnO | 3.60 | 5.61×10^3^ | S10 |
| ZTO | 3.83 | 3.61 | S11 |
| Ga_2_O_3_ | 4.98 | 0.07 | S12 |
| Si:Ga_2_O_3_ | 4.8 | 0.15 | S13 |
| Sn:Ga_2_O_3_ | 5.83 | 5×10^4^ | S14 |
| Ga_2_O_3_:CdO | 4.05 | 0.40 | S15 |
| GTO | 3.78 | 2.60 | S16 |
| GTO | 3.55 | 5.10 | S16 |
| GZO | 3.26 | 20.56 | S17 |
| GZO | 3.26 | 5.7 | S18 |
| GZO | 3.26 | 2.30×10^2^ | S19 |
| GZO | 4.20 | 1 | S9 |
| GZTO | 3.90 | 4.84 | S11 |
| GZTO | 3.44 | 0.68 | S20 |
| In_2_Mg_8_O | 3.93 | 2.02 | This work |
| In_4_Mg_6_O | 4.40 | 1.15 | This work |
| In_5_Mg_5_O | 4.70 | 0.55 | This work |
| In_6_Mg_4_O | 4.98 | 0.34 | This work |
| In_8_Mg_2_O | 5.43 | 0.17 | This work |

ZTO = ZnSnO

GTO = GaSnO

GZO = GaZnO

GZTO = GaZnSnO

**Table S2. Summary of oxide-based synaptic devices’ energy consumption.**

| Material | Energy Consumption | Reference |
| --- | --- | --- |
| Ga_2_O_3_ | 71 fJ  (0.6 mV×0.67 nA×180 ms) | S42 |
| Ga_2_O_3_ | 11 fJ  (200 mV×0.22 nA×100 ms) | S43 |
| GaO_x_ | 14 pJ  (2 V×0.07 nA×100 ms) | S44 |
| Ga_2_O_3_/MgO | 1.08 nJ  (200 mV×0.22 nA×100 ms) | S45 |
| ITO/Ga_2_O_3_ | 12 pJ  (10 mV×1.24 nA×1 s) | S46 |
| IGZO | 15 pJ  (1 V×1.5 pA×10 s) | S47 |
| IGZO | 21nJ  (10 V×4.46 nA×500 ms) | S48 |
| IGZO | 8.6 pJ  （0.1 V×4.3 nA×20 ms） | S49 |
| IGZO | 1.825 fJ  (1 mV×36.5 pA×50 ms) | S50 |
| IZO/IGZO | 0.269 fJ  (3 mV×89.67 pA×1 ms) | S51 |
| IZO NWs | 78.75 fJ  (0.5 mV×3.1499 nA×50 ms) | S52 |
| WO_3-x_ | 20 fJ(Inference)  3.5 pJ (Training) | S53 |
| ZnO/ITO/WO_3-x_ | 90 nJ  （0.4 V×22.5 nA×10 s） | S54 |
| InAlZnO | 75 fJ  (1 V×15 pA×5 ms) | S55 |
| ZnMgO | 2.34 pJ | S56 |
| TaO_y_/NP-TaO_x_ | 9 nJ | S57 |
| TaO_x_/HfO_x_ | 10 fJ/2.55 pJ (1-cell/1-bit operation) | S58 |
| Y:HfO_2_ | 0.3575 pJ  (0.1 mV×7.15 nA×0.5 s) | S59 |
| SiO_2_/Hf_0.5_Zr_0.5_O_2_ | 0.12 fJ | S60 |
| HZO | 1.96 nJ(Training) | S61 |
| HZO/n-STO | 0.1 fJ  (1 V×0.1 pA×1 ms) | S62 |
| Co_3_O_4_ nanoflowers | 74 pJ | S63 |
| FAPbI_3_/SnO_2_ | 17.5 pJ  (0.7 V×50 nA×0.5 ms) | S64 |
| In_5_Mg_5_O | 2.1 fJ  (10 μV×0.7 nA×0.3 ms) | This work |

IZO = InZnO

HZO = Hf_0.5_Zr_0.5_O_2_

Movie S1: DUV-LALED array for noise-filtering display with channel length of 60 μm

Movie S2: DUV-LALED array for noise-filtering display with channel length of 120 μm

Movie S3: DUV-LALED array for noise-filtering display with channel length of 240 μm

**References**

S1. X. Li, Z. Li, J. Hu, et al., “Tunneling-Barrier-Controlled Sensitive Deep Ultraviolet Photodetectors Based on Van Der Waals Heterostructures,” *Nature Communications* 16 (2025): 2209, https://doi.org/10.1038/s41467-025-56886-8.

S2. Z. Wu, L. Ye, Z. Lin, et al., “Synergistic Dual Built-In Electric Fields and HfO_2_ Band Engineering for Self-Powered Solar-Blind UV Detectors on Silicon,” *Applied Physics Letters* 127 (2025): 153305, https://doi.org/10.1063/5.0290624.

S3. T.-S. Lin and C.-T. Lee, “Performance Investigation of *P-I-N* ZnO-Based Thin Film Homojunction Ultraviolet Photodetectors,” *Applied Physics Letters* 101 (2012): 221118, https://doi.org/10.1063/1.4768786.

S4. X. Zhao, J. Dai, J. Dong, et al., “Lead-Free Perovskite/ZnO Heterostructure for Flexible Visible-Blind Ultraviolet Photodetection,” *Applied Physics Letters* 124 (2024): 253301, https://doi.org/10.1063/5.0210954.

S5. B. Zhao, F. Wang, H. Chen, et al., “An Ultrahigh Responsivity (9.7 mA W^-1^) Self-Powered Solar-Blind Photodetector Based on Individual ZnO-Ga_2_O_3_ Heterostructures,” *Advanced Functional Materials* 27 no. 17 (2017): 1700264, https://doi.org/10.1002/adfm.201700264.

S6. F. Cao, E. Hong, Z. Li, S. Han and X. Fang, “Real-Time UVC Imaging and High Safety Communication Based on Dual-Detectable Lead-Free Perovskite Heterostructure,” *Advanced Functional Materials* 35 no. 24 (2025): 2422161, <https://doi.org/10.1002/adfm.202422161>.

S7. W. J. Park, H. S. Shin, B. D. Ahn, et al., “Investigation on Doping Dependency of Solution-Processed Ga-doped ZnO Thin Film Transistor,” *Applied Physics Letters* 93 (2008): 083508, <https://doi.org/10.1063/1.2976309.>

S8. T. S. Kang, T. Y. Kim, G. M. Lee, H. C. Sohnb, and J. P. Hong, “Highly Stable Solution-processed ZnO Thin Film Transistors Prepared via a Simple Al Evaporation Process,” *Journal of Materials Chemistry C* 2 (2014): 1390-1395, <https://doi.org/10.1039/c3tc32341b.>

S9. A. Zeumault and V. Subramanian, “Use of High-k Encapsulation to Improve Mobility in Trap-limited Metal-oxide Semiconductors,” *physica status solidi (b)* 254 no. 10 (2017): 1700124, <https://doi.org/10.1002/pssb.201700124.>

S10. Y. Caglar, M. Caglar, S. Ilican, S. Aksoy, and F. Yakuphanoglu, “Effect of Channel Thickness on the Field Effect Mobility of ZnO-TFT Fabricated by Sol Gel Process,” *Journal of Alloys and Compounds* 621 (2015): 189-193, <http://doi.org/10.1016/j.jallcom.2014.09.190.>

S11. B. D. Ahn, H. J. Jeon and J.-S. Park, “Effects of Ga:N Addition on the Electrical Performance of Zinc Tin Oxide Thin Film Transistor by Solution-Processing,” *ACS Applied Materials & Interfaces* 6 (2014): 9228-9235, <https://doi.org/10.1021/am5013672.>

S12. Y. Wang, Y. Xue, J. Su, et al., “Realization of Cost-effective and High-performance Solar-blind Ultraviolet Photodetectors Based on Amorphous Ga_2_O_3_ Prepared at Room Temperature,” *Materials Today Advances* 16 (2022): 100324, <https://doi.org/10.1016/j.mtadv.2022.100324>.

S13. L. Shen, X. Pan, T. Zhang, et al., “Improved β‑Ga_2_O_3_ Solar‑Blind Deep‑Ultraviolet Thin‑Film Transistor Based on Si‑Doping,” *Journal of Electronic Materials* 51 (2022): 3579-3588, <https://doi.org/10.1007/s11664-022-09599-3.>

S14. Y. Yoon, M. J. Kim, B. J. Cho, M. Shin, and W. S. Hwang, “An 8-nm-thick Sn-doped Polycrystalline β-Ga_2_O_3_ MOSFET with a “normally off” operation,” *Applied Physics Letters* 119 (2021): 122103, <https://doi.org/10.1063/5.0065244.>

S15. X. Xiao, L. Liang, Y. Pei, et al., “Solution-processed Amorphous Ga_2_O_3_:CdO TFT-type Deep UV Photodetectors,” *Applied Physics Letters* 116 (2020): 192102, <https://doi.org/10.1063/5.0007617.>

S16. C. -Y. Tsay and S. -C. Liang, “Ultraviolet-assisted Annealing for Low-temperature Solution-processed P-type Gallium Tin Oxide (GTO) Transparent Semiconductor Thin Films,” *Materials Science in Semiconductor Processing* 71 (2017): 441-446, http://doi.org/10.1016/j.mssp.2017.09.008.

S17. D. Han, S. Zhang, F. Zhao, et al., “Transparent Gallium Doped Zinc Oxide Thin-film Transistors Fabricated on Glass Substrate,” *Thin Solid Films* 594 (2015): 266-269, http://doi.org/10.1016/j.tsf.2015.06.026.

S18. V. P. Verma, D. -H. Kim, H. Jeon, M. Jeon, and W. Choi, “Characteristics of Low Doped Gallium-zinc Oxide Thin Film Transistors and Effect of Annealing Under High Vacuum,” *Thin Solid Films* 516 (2008): 8736-8739, <http://doi.org/10.1016/j.tsf.2008.06.054.>

S19. H. Jeno, V. P. Verma, S. Hwang, et al., “Characteristics of Gallium-doped Zinc Oxide Thin-Film Transistors Fabricated at Room Temperature Using Radio Frequency Magnetron Sputtering Method,” *Japanese Journal of Applied Physics* 47 no. 1 (2008): 87-90, <http://doi.org/10.1143/JJAP.47.87.>

S20. J. H. Lee, C. H. Kim and H. S. Kim, “Stability Characteristics of Gallium-doped Zinc-tin-oxide Thin-film Transistors Fabricated Using a Sol-gel Method,” *Journal of the Korean Physical Society* 62 no. 8 (2013): 1176-1182, <http://doi.org/10.3938/jkps.62.1176.>

S21. J. H. Park, D. Y. Kim, E. F. Schubert, J. Cho and J. K. Kim, “Fundamental Limitations of Wide-Bandgap Semiconductors for Light-Emitting Diodes,” *ACS Energy Letters* 3 no. 3 (2018): 655-662, <https://doi.org/10.1021/acsenergylett.8b00002>.

S22. W. A. Harrison 2012, “Chapter 2-A-The Energy Bands.” In *Electronics Structure and Properties of Solids*. First ED., edited by W. A. Harrison, Newburyport : Dover Publications. ISBN 978-0486660219.

S23. C. G. Van de Walle and R. M. Martin, “Theoretical Calculations of Semiconductor Heterojunction Discontinuities,” *Journal of Vacuum Science & Technology B* 4, no. 4 (1986): 1055-1059, https://doi.org/10.1116/1.583580.

S24. P. Hohenberg and W. Kohn, “Inhomogeneous Electron Gas,” *Physical Review B* 136 (1964): B864-B871, https://doi.org/10.1103/PhysRevB.34.5621.

S25. J. Varignon, M. Bibes and A. Zunger, “Origin of Band Gaps in 3d Perovskite Oxides,” *Nature Communications* 10 (2019): 1658, https://doi.org/10.1038/s41467-019-09698-6.

S26. X. Shao, L. Paetow, M. E. Tuckerman and M. Pavanello, “Machine Learning Electronic Structure Methods Based on the One-electron Reduced Density Matrix,” *Nature Communications* 14 (2023): 6281, https://doi.org/10.48550/arXiv.2302.10741.

S27. Y.-C. Chen, T. Peterson, Q. Jia, et al., “Large and Tunable Electron-depletion-based Voltage-controlled Magnetic Anisotropy in the CoFeB/MgO System via Work-function-engineered Pt*_x_*W_1–_*_x_* Underlayers,” *ACS Nano* 19, no. 16 (2025): 15953-15962, https://doi.org/10.1021/acsnano.5c01956.

S28. T. V. Perevalov, D. R. Islamov, T. M. Zalyalov, et al., “Electron and Hole Bipolar Injection in Magnesium Oxide Films,” *Applied Physics Letters* 124, no. 4 (2024): 042903, https://doi.org/10.1063/5.0180827.

S29. A. Islam, E. Huang, Y. Tian, et al., “Low-temperature Activation and Coupling of Methane on MgO Nanostructures Embedded in Cu_2_O/Cu (111),” *ACS Nano* 18, no. 41 (2024): 28371-28381, https://doi.org/10.1021/acsnano.4c10811.

S30. J. K. Saha and J. Jang, “Saturation Mobility of 100 cm^2^ V^–1^ s^–1^ in ZnO Thin-film Transistors through Quantum Confinement by a Nanoscale In_2_O_3_ Interlayer Using Spray Pyrolysis,” *ACS Nano* 18, no. 44 (2024): 30484-30496, https://doi.org/10.1021/acsnano.4c08644.

S31. J. Zhang, A. Charnas, Z. Lin, et al., “Fluorine-passivated In_2_O_3_ Thin Film Transistors with Improved Electrical Performance via Low-temperature CF_4_/N_2_O Plasma,” *Applied Physics Letters* 121, no. 17 (2022): 172101, https://doi.org/10.1063/5.0113015.

S32. Y. S. Liu, C. I. Hsieh, Y. J. Wu, Y. S. Wei, P. M. Lee and C. Y. Liu, “Transparent p-type AlN:SnO_2_ and p-AlN:SnO_2_/n-SnO_2_:In_2_O_3_ p-n Junction Fabrication,” *Applied Physics Letters* 101, no. 12 (2012): 122107, https://doi.org/10.1063/1.4754134.

S33. D. A. Shirley, “High-resolution X-Ray Photoemission Spectrum of the Valence Bands of Gold,” *Physical Review B* 5 (1972): 4709, https://doi.org/10.1103/PhysRevB.5.4709.

S34. K. Liu, M. Sakurai, M. Aono and D. Shen, “Ultrahigh-gain Single SnO_2_ Microrod Photoconductor on Flexible Substrate with Fast Recovery Speed,” *Advanced Functional Materials* 25, no. 21 (2015): 3157-3163, https://doi.org/10.1002/adfm.201500231.

S35. P. Wu and J. Kong, “Doping for Ohmic Contacts in 2D Transistors,” *Nature Electronics* 7 (2024): 519-520, https://doi.org/10.1038/s41928-024-01191-3.

S36. K. Uryu, Y. Deng, T. Nanjo and T.-K. Suzuki, “Low-temperature-annealed Ohmic Contacts to Ultrathin-AlGaN/GaN Heterostructures with No Two-dimensional Electron Gas,” *Applied Physics Letters* 126, no. 23 (2025): 232103, https://doi.org/10.1063/5.0260035.

S37. H. Du, Z. Liu, L. Hao, et al., “Low Ohmic Contact Resistivity Realized by *in situ* SiN_x_ Insertion for High Al-composition-AlGaN/GaN Heterostructure,” *Applied Physics Letters* 121, no. 17 (2022): 172102, https://doi.org/10.1063/5.0100329.

S38. Y. Cui, L. Cheng, B. Cheng, et al., “Saturated Salt Hydrogel Engineering for Tunable ZnO Ultraviolet Sensor Performance and Mitigated Humidity Interference,” *ACS Applied Materials & Interfaces* 18, no. 2 (2026): 4015-4024, https://doi.org/10.1021/acsami.5c19411.

S39. Y. Li, F. D. Valle, M. Simonnet, I. Yamada and J.-J. Delaunay, “Competitive Surface Effects of Oxygen and Water on UV Photoresponse of ZnO Nanowires,” *Applied Physics Letters* 94, no. 2 (2009): 023110, https://doi.org/10.1063/1.3073042.

S40. G. Dubourg, M. Radović and B. Vasić, “Laser-Tunable Printed ZnO Nanoparticles for Paper-Based UV Sensors with Reduced Humidity Interference,” *Nanomaterials* 11, no 1 (2021): 80, https://doi.org/10.3390/nano11010080.

S41. Y. M. Juan, S. J. Chang, H. T. Hsueh, et al., “Eﬀects of Humidity and Ultraviolet Characteristics on β-Ga_2_O_3_ Nanowire Sensor,” *RSC Advances* 5 (2015): 84776-84781, <https://doi.org/10.1039/C5RA16710H>.

S42. A. Kaur, S. Sikdar, S. K. Yadav, and S. Dhar, “PLD-grown Semi-insulating Ga_2_O_3_ Thin Film-Based Optoelectronic Artificial Synaptic Devices for Neuromorphic Computing Applications,” *Advanced Materials Technologies* 9 (2024): 2400464, <https://doi.org/10.1002/admt.202400464.>

S43. R. Zhu, H. Liang, S. Hu, Y. Wang, and Z. Mei, “Amorphous-Ga_2_O_3_ Optoelectronic Synapses with Ultra-low Energy Consumption,” *Advanced Electronic Materials* 8 (2022): 2100741, <https://doi.org/10.1002/aelm.202100741.>

S44. S. Wang, C. He, J. Tang, R. Yang, D. Shi, and G. Zhang, “Electronic Synapses Based on Ultrathin Quasi-two-dimensional Gallium Oxide Memristor,” *Chinese Physics B* 28 no. 1 (2019): 017304, <https://doi.org/10.1088/1674-1056/28/1/017304.>

S45. X. Chen, W. Mi, Meng Li, et al., “Optoelectronic Artificial Synapses Based on β-Ga_2_O_3_ Films by RF Magnetron Sputtering,” *Vacuum* 192 (2021): 110422, <https://doi.org/10.1016/j.vacuum.2021.110422.>

S46. R. Li, Y. Lin, Y. Li, et al., “Amorphous Gallium Oxide Homojunction-based Optoelectronic Synapse for Multi-functional Signal Processing,” *Journal of Semiconductors* 44 (2023): 074101, https://doi.org/10.1088/1674-4926/44/7/074101.

S47. J. J. Yu, L. Y. Liang, L. X. Hu, et al., “Optoelectronic Neuromorphic Thin-film Transistors Capable of Selective Attention and with Ultra-low Power Dissipation,” *Nano Energy* 62 (2019): 772-780, <https://doi.org/10.1016/j.nanoen.2019.06.007.>

S48. M. Lee, S. Song, and Y.-H. Kim, “Emulation of synaptic behaviors using amorphous indium-gallium-zinc-oxide-based photoelectric synaptic devices,” *Japanese Journal of Applied Physics* 58 (2019): 090607, <https://doi.org/10.7567/1347-4065/ab2c2d.>

S49. X. Lin, H. Long, Shuo Ke, et al., “Indium-Gallium-Zinc-Oxide-Based Photoelectric Neuromorphic Transistors for Spiking Morse Coding,” *Chinese Physics Letters* 39 (2022): 068501, <https://doi.org/10.1088/0256-307X/39/6/068501.>

S50. L. Zhu, S. Li, J. Lin, et al., “Ultra-low Power IGZO Optoelectronic Synaptic Transistors for Neuromorphic Computing,” *Science China Information Sciences* 67 no. 12 (2024): 222401, <https://doi.org/10.1007/s11432-023-3966-8.>

S51. S. -I. Cho, J. B. Jeon, J. H. Kim, et al., “Synaptic Transistors with Human Brain-like fJ Energy Consumption via Double Oxide Semiconductor Engineering for Neuromorphic Electronics,” *Journal of Materials Chemistry C* 9 (2021): 10243, <https://doi.org/10.1039/d1tc01451j.>

S52. L. Liu, B. Cui, W. Xu, Y. Ni, S. Zhang, and W. Xu, “Highly Aligned Indium Zinc Oxide Nanowire-based Artificial Synapses with Low-energy Consumption,” *Journal of Industrial and Engineering Chemistry* 88 (2020): 111-116, <https://doi.org/10.1016/j.jiec.2020.03.030.>

S53. R. Yang, K. Terabe, Y. Yao, et al., “Synaptic Plasticity and Memory Functions Achieved in a WO_3−x_-based Nanoionics Device by Using the Principle of Atomic Switch Operation,” *Nanotechnology* 24 (2013): 384003, <https://doi.org/10.1088/0957-4484/24/38/384003.>

S54. J. Pan, T. Wu, W. Yang, Y. Li, J. Zhang and H. Kan, “ZnO-ITO/WO_3-x_ Heterojunction Structured Memristor for Optoelectronic Co-modulation Neuromorphic Computation,” *Science China Mater* 67 no. 9 (2024): 2838-2847, <https://doi.org/10.1007/s40843-024-2946-y.>

S55. W. Xiao, Y. Dong, R. Ci, G. Liu, and F. Shan, “Oxygen Vacancy-Induced Synaptic Plasticity in InAlZnO Nanofiber Transistors for Low-Power Neuromorphic Electronics,” *IEEE Transactions on Electron Devices* 71 no. 10 (2024): 6430-6434, <https://doi.org/10.1109/TED.2024.3449828.>

S56. J. Li, K. Chen, C. Wu, et al., “Low-power Optoelectronic Synaptic Devices with ZnMgO in Deep-ultraviolet Encryption Computation,” *Applied Physics Letters* 126 (2025): 222104, <https://doi.org/10.1063/5.0268617.>

S57. S. Choi, S. Jang, J. H. Moon, et al., “A Self-rectifying TaOy/Nanoporous TaOx Memristor Synaptic Array for Learning and Energy-efficient Neuromorphic Systems,” *npg asia materials* 10 (2018): 1097-1106, <https://doi.org/10.1038/s41427-018-0101-y.>

S58. P. Yao, H. Wu, B. Gao, et al., “Fully hardware-implemented memristor convolutional neural network,” *Nature* 577 (2020): 641-646, <https://doi.org/10.1038/s41586-020-1942-4.>

S59. Z. -L. Tong, Y. -P. Jiang, M. Huang, et al., “Optoelectronic Synaptic Properties of Y-doped HfO_2_ Ferroelectric Memristor for Neuromorphic Computing,” *Physica B: Condensed Matter* 714 (2025): 417412, <https://doi.org/10.1016/j.physb.2025.417412.>

S60. H. Wang, Z. Guan, J. Li, et al., “Silicon-Compatible Ferroelectric Tunnel Junctions with a SiO_2_/Hf_0.5_Zr_0.5_O_2_ Composite Barrier as Low-voltage and Ultra-high-speed Memristors,” *Advanced Materials* 36 (2024): 2211305, <https://doi.org/10.1002/adma.202211305.>

S61. Y. Zhang, S. Yan, Y. Zhu, et al., “Flexible Zr‑doped Hafnium Oxide Ferroelectric Memcapacitive Synaptic Devices for Neuromorphic Computing,” *Advanced Composites and Hybrid Materials* 8 (2025): 278, <https://doi.org/10.1007/s42114-025-01364-4.>

S62. N. Siannas, C. Zacharaki, P. Tsipas, et al., “Electronic Synapses Enabled by an Epitaxial SrTiO_3-δ_/Hf_0.5_Zr_0.5_O_2_ Ferroelectric Field-eﬀect Memristor Integrated on Silicon,” *Advanced Functional Materials* 34 (2024): 2311767, <https://doi.org/10.1002/adfm.202311767.>

S63. Y. Li, X. Zhou, J. Guo, et al., “Artificial Synapse Based on Co_3_O_4_ Nanoflowers with Picojoule Energy Consumption, Microsecond Time Constant, and Visual Learning Capability,” *Japanese Journal of Applied Physics* 64 (2025): 080906, <https://doi.org/10.35848/1347-4065/adf8a7.>

S64. S. -U. Lee, S. -Y. Kim, J. -H. Lee, et al., “Artificial Synapse Based on a δ‑FAPbI_3_/Atomic-layer-deposited SnO_2_ Bilayer Memristor,” *Nano Letters* 24 (2024): 4869-4876, https://doi.org/10.1021/acs.nanolett.4c00253.
